# Explainable spatiotemporal Graph Neural Networks: Multi-City PM_2.5_ forecasting in Bangladesh with geographic graph topology validation

**DOI:** 10.1371/journal.pone.0351928

**Published:** 2026-10-12

**Authors:** Pritom Ray Nobin

**Affiliations:** Department of Urban and Regional Planning, Khulna University of Engineering & Technology (KUET), Khulna, Bangladesh; Beijing Institute of Technology, CHINA

## Abstract

Accurate forecasting of fine particulate matter (PM_2.5_) is critical for mitigating severe health risks in South Asia, yet it remains challenging in data-scarce regions like Bangladesh. While spatiotemporal graph neural networks (GNNs) offer a promising solution by capturing atmospheric transport, their effectiveness and the physical validity of their assumed spatial graphs have rarely been rigorously tested in such constrained settings. This study addresses this gap by evaluating a graph diffusion gated recurrent unit (GD-GRU) model using four years of daily, multi-source environmental data across a 12-city network. For forecasts up to three days ahead, the GD-GRU achieves an overall mean absolute error of 8.853 µg/m^3^ and an R^2^ of 0.666, significantly outperforming non-graph baselines under FDR-corrected Diebold–Mariano testing (p < 0.001). Furthermore, the model significantly outperforms DCRNN when pooled across all forecast horizons (p < 0.001), while remaining closely matched at individual lead times. Crucially, empirical validation of the model’s underlying spatial assumptions demonstrates that occlusion-based spatial influence closely tracks the fixed geographic adjacency (r = 0.928, p < 0.001), and this simple distance-based graph outperforms unconstrained adaptive, wind-informed, and hybrid alternatives. Finally, a multi-method explainability framework shows strong rank convergence (Spearman ρ = 0.590 to 0.783), identifying the Green Leaf Index, wind speed, atmospheric pressure, and seasonal encoding as the dominant predictors of PM_2.5_. These findings confirm that carefully validated, interpretable static graph models can deliver robust air quality forecasts without requiring dense sensor networks, offering a practical template for environmental monitoring in data-limited regions.

## Introduction

Fine particulate matter with an aerodynamic diameter of 2.5 µm or less (PM_2.5_) is the most important environmental risk factor for premature death worldwide. It is responsible for an estimated 4.14 million annual deaths from ambient PM_2.5_ exposure [1]. South Asia bears a disproportionate share of this burden. In 2023, Bangladesh recorded the world’s highest national average PM_2.5_ concentration at 79.9 μg/m^3^, more than fifteen times the WHO guideline and twice its national standard [2]. Long-term exposure to ambient PM_2.5_ is estimated to cause about 173,000 premature deaths each year in Bangladesh, which represents roughly 20% of total mortality in the country [1,3]. The pollution environment in Bangladesh is also highly variable across regions. Brick kilns concentrated in the northwest produce severe pollution during the dry season. Along the southern coast, maritime boundary-layer conditions create a different seasonal pollution pattern. In northern cities, PM_2.5_ levels often rise between November and March because of pollution transported by northwesterly winds from the Indo-Gangetic Plain [4,5]. These combined factors make Bangladesh a particularly challenging environment for PM_2.5_ forecasting, given the diversity of emission sources and the sparse monitoring infrastructure.

### From numerical models to spatiotemporal deep learning

Physics-based chemical transport models such as CMAQ and WRF-Chem can produce physically interpretable air quality forecasts. However, they require detailed emission inventories and boundary conditions that are not available at the national scale in Bangladesh [6]. Classical machine learning approaches, such as random forests and gradient-boosted trees, are easier to apply in data-limited regions and remain strong non-deep-learning baselines for tabular air-quality prediction tasks [7]. However, they have key structural limitations. These models treat each monitoring station independently, ignoring the physical reality that pollution transported by wind can affect downwind cities after a short delay. In Bangladesh, for example, pollution from the Indo-Gangetic Plain during the dry season contributes substantially to PM_2.5_ variation in northern cities, a pattern that station-independent models fail to capture [4,8].

Recurrent deep learning models, such as Long Short-Term Memory (LSTM) and Gated Recurrent Unit (GRU) networks, capture temporal dynamics by learning nonlinear relationships from past observations. These models have consistently outperformed classical approaches for single-station PM_2.5_ prediction [9,10]. However, they mainly capture short-term temporal dependencies and fail to model long-term patterns effectively. Transformer-based models address long-range temporal dependencies using self-attention, but they typically require large training datasets and are less suitable for monitoring networks with limited samples. However, all of these approaches mainly model temporal patterns. They do not explicitly represent the spatial movement of pollution, which is an important part of real atmospheric behavior [11,12].

### Spatiotemporal graph neural networks for air quality

Graph Neural Networks (GNNs) provide a natural framework for this problem. In this approach, each monitoring station is treated as a node, atmospheric transport pathways as weighted edges, and graph convolution allows pollution signals to move across the network in a way that reflects advection–diffusion processes in the atmosphere. Early GNN studies for air quality prediction combined Graph Convolutional Networks with Long Short-Term Memory encoders to forecast PM_2.5_ across multiple monitoring stations, showing clear accuracy improvements compared with models that treat each station independently [11]. Later work demonstrated that the way the graph is constructed is critically important. For example, the PM_2.5_-GNN model introduced by [13] incorporated geographic and meteorological information when defining graph connections, which led to better performance than models that attempted to learn adjacency purely from data. Diffusion Convolutional Recurrent Neural Network introduced an important innovation by replacing standard linear operations inside Gated Recurrent Unit cells with diffusion-based graph convolutions [14]. This design allows the reset and update gates of the recurrent unit to include information from neighboring nodes at each time step. As a result, the memory and update behavior of each city can depend on the current pollution levels of nearby cities. Studies show that this approach performs better than architectures that apply graph convolution only to the input features before passing them to a standard recurrent model [14,15]. The utility of graph-augmented recurrent architectures extends beyond atmospheric modeling, with GNN and GRU frameworks demonstrating strong performance in diverse spatiotemporal applications, including flash flood forecasting, traffic accident severity prediction, and lithium-ion battery state-of-health and remaining-useful-life prediction [16–19]. These applications highlight the capacity of spatiotemporal GNNs to capture complex physical and degradation processes across diverse network structures.

More recent work has explored attention-based and dynamic graph approaches. Models based on Graph Attention Networks learn adaptive weights for each edge in the graph, allowing the model to adjust the importance of connections between stations [20]. Other studies use dynamic graphs whose edge weights change with meteorological conditions, which helps capture seasonal wind shifts that can alter pollution transport pathways [21,22]. In regions with dense monitoring networks, typically 30–200 stations in countries such as China or the United States, these models commonly report +1-day PM_2.5_ prediction R^2^ values between 0.75 and 0.88 [22,23].

### The data-scarcity problem and graph construction

The transferability of these advances to developing-country monitoring networks is still uncertain. Most spatiotemporal deep learning studies for air quality have been conducted on dense monitoring systems where hundreds of stations provide strong relational signals for learning spatial dependencies [22]. In those settings, dynamic or adaptive graph structures can effectively use the large amount of spatial information available. In contrast, Bangladesh operates only a very small monitoring network, and the available training data consist of a limited number of observation sequences. Under such conditions, fully learnable graph structures may easily fit spatial correlations that come from sampling noise rather than real atmospheric transport. In this kind of data-limited network, the key question is not which spatial representation is most expressive, but which one is most reliable given the available data [24].

This limitation motivates a different design strategy. Instead of learning the spatial graph entirely from data, the graph can be built using geographic knowledge and known regional atmospheric patterns, then kept fixed during training. Model capacity can then focus mainly on learning temporal patterns and feature relationships. However, an important uncertainty remains regarding whether a fixed geographic graph truly represents the spatial influence patterns learned by the model, or whether it simply imposes a convenient assumption. Previous studies have not directly examined this issue. Addressing it requires post hoc spatial analysis that compares the fixed graph structure with the behavior learned by the model [25]. This study directly addresses that question. Using post hoc attribution analysis, we empirically test whether a static geographic adjacency reflects the spatial influence structure that a model learns from data, an aspect that has received limited attention in previous atmospheric monitoring studies.

### Explainability as a validation and trust requirement

Another limitation of existing spatiotemporal GNN studies is the lack of detailed explainability analysis. Most research reports overall accuracy metrics but does not examine which features directly influence predictions, whether the model uses meaningful temporal memory, or whether the graph structure reflects real atmospheric relationships. For applications in public health and environmental policy, this lack of transparency creates a serious trust problem. Forecasts that cannot be explained in physically meaningful terms are difficult for regulators and health authorities to rely on [24,26,27].

Several explainability methods have been applied individually to air-quality models, including gradient-based saliency, permutation feature importance, and SHAP values [27]. However, each method has its own limitations. Gradient methods can be sensitive to input scaling, permutation methods may misinterpret correlated variables, and SHAP estimates depend on assumptions used during integration. To our knowledge, no previous spatiotemporal GNN study for air quality has used a combined explainability framework that compares multiple attribution methods for consistency. When different methods with distinct assumptions produce similar importance rankings, the result provides much stronger evidence that the identified features truly influence the model’s predictions [28,29].

### Research gaps and contributions

Four main gaps motivate this study. First, no previous research has applied a spatiotemporal GNN to multi-city, multi-step PM_2.5_ forecasting in Bangladesh or in any similarly data-limited monitoring network in South Asia. Existing studies focused on Bangladesh are limited to single-station estimation in Dhaka [30] or simple time-series models that do not produce multi-step forecasts across a national monitoring network. Second, the reliability of static geographic graphs as a representation of spatial influence has not been tested through post hoc attribution analysis in atmospheric monitoring studies. Third, convergent explainability frameworks that combine several attribution methods have not been applied to spatiotemporal GNN models for air-quality forecasting. Fourth, the temporal memory of GRU-based spatiotemporal encoders, meaning how far back the model uses past observations for different forecast horizons, has not been systematically studied using gradient sensitivity analysis.

This paper addresses these gaps through the following contributions:

1. **Graph topology validation.** Using occlusion-based spatial influence analysis, we provide empirical post hoc evidence that a static distance-based geographic adjacency approximates the spatial influence structure learned by a spatiotemporal GNN in a data-limited monitoring network, supported by a strong Pearson correlation (r = 0.928, p < 0.001) between the learned influence matrix and the fixed geographic adjacency.
2. **Application in a sparse monitoring network.** We present the first spatiotemporal GNN applied to multi-city PM_2.5_ forecasting across Bangladesh’s 12-city monitoring network, demonstrating that useful multi-step forecasts are achievable under conditions far sparser than those used in comparable studies from China, the United States, or Europe.
3. **Explainability framework.** We implement a multi-method explainability framework that combines Gradient × Input saliency, temporal sensitivity analysis, permutation feature importance, KernelSHAP, and occlusion-based spatial influence analysis. This approach produces interpretable results for feature importance, temporal memory, and spatial influence within the model and ensures robust evidence of genuine feature importance rather than method-specific artifacts.
4. **Model implementation.** We implement GD-GRU, a Chebyshev graph diffusion GRU ensemble, as the framework for these analyses. A direct comparison with DCRNN under identical experimental conditions shows a statistically significant advantage for GD-GRU across pooled forecast horizons (DM test, p < 0.001), despite closely matched performance at individual horizons. This suggests that, in sparse networks, the choice of diffusion operator may be less important than shared design factors such as graph construction and regularization, while GD-GRU’s symmetric formulation and faster training provide additional practical advantages.

## Methodology

### Study area

Bangladesh is a low-lying deltaic country in South Asia (20.67°N–26.63°N, 88.01°E–92.67°E), bordered by India on three sides and the Bay of Bengal to the south. Despite its small land area, it supports a population of about 170 million, making it one of the most densely populated countries in the world. Much of this population is concentrated in urban areas, leading to high and uneven air pollution levels [3]. Air quality in Bangladesh is influenced by three main factors: local emissions, regional weather patterns, and pollution transported from the Indo-Gangetic Plain [4]. The Bangladesh Department of Environment operates a Continuous Air Monitoring Station network across major cities. Twelve cities with continuous PM_2.5_ data are used in this study: Barisal, Bogra, Comilla, Dhaka, Feni, Jashore, Khulna, Mymensingh, Narsingdi, Pabna, Rajshahi, and Sylhet (Fig 1). The spatial pattern of these cities can be grouped into three clusters based on geography and atmospheric conditions. The southern coastal cities, Barisal, Khulna, and Jashore, are located close to the Bay of Bengal and experience steady maritime airflow, which leads to more stable boundary-layer conditions and fewer sudden pollution events [31]. The north-central cities, Bogra, Mymensingh, Pabna, Narsingdi, and Rajshahi, are situated in the Ganges–Brahmaputra floodplain and are influenced by both transboundary pollution from the Indo-Gangetic Plain during the dry season and strong local emissions from brick kilns [4,8]. The eastern cities, Dhaka, Comilla, Feni, and Sylhet, include the central industrial corridor and the northeastern highlands. Dhaka has high emissions from traffic and industry throughout the year, while Sylhet’s location near the Khasi and Jaintia hills reduces its exposure to incoming pollution and results in lower variability [31].

**Fig 1:**
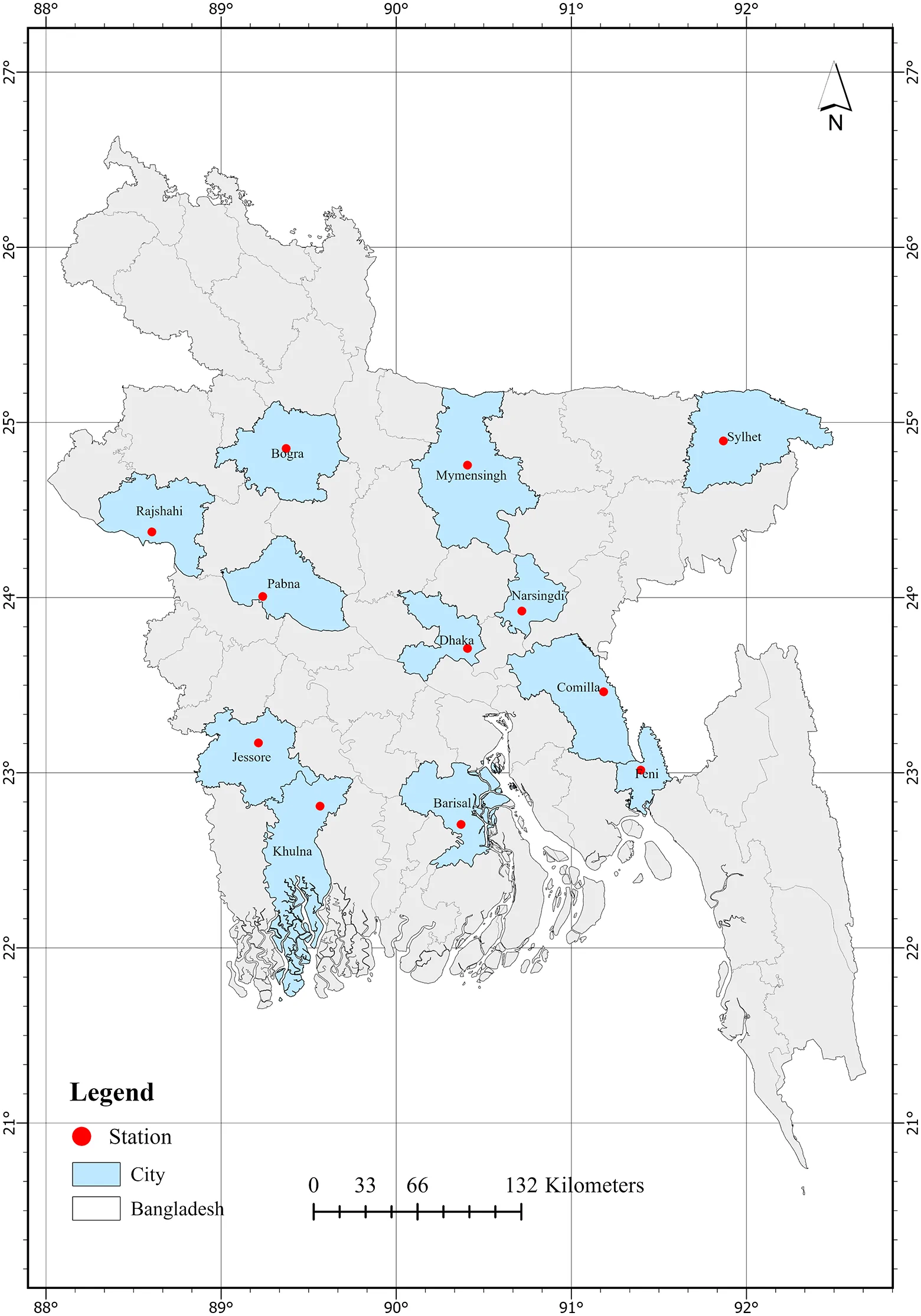
Study area map showing the 12-city PM_2.5_ monitoring network in Bangladesh. Administrative boundaries sourced from the Bangladesh Agricultural Research Council, CC BY 4.0.

### Data sources and feature construction

The modelling dataset combines observations and remote sensing products from seven sources for 12 cities in Bangladesh between 2022 and 2025. Table 1 lists all features.

**Table 1:** Features input in model.

Feature	Description	Unit	Temporal Resolution	Source
PM_2.5_	Daily mean PM_2.5_ concentration (Target Variable)	µg m ^−^ ³	Daily	Bangladesh CAMS
temperature	Daily mean 2-metre air temperature	°C	Daily	ERA5
humidity	Daily mean relative humidity at 2 m	%	Daily	ERA5
rainfall_sum	Total daily precipitation	mm	Daily	ERA5
pressure	Daily mean surface pressure	hPa	Daily	ERA5
wind_speed	Daily mean 10-metre wind speed	m s ^−^ ¹	Daily	ERA5
wind_direction	Daily circular mean wind direction at 10 m	degrees	Daily	ERA5
solar_rad_sum	Daily accumulated shortwave radiation	J/m^2^	Daily	ERA5
NDVI	Normalised Difference Vegetation Index: (NIR − R)/(NIR + R)	dimensionless	8-day	MODIS
GARI	Green Atmospherically Resistant Index: (NIR−(G−(B − R)))/(NIR+(G−(B − R)))	dimensionless	8-day	MODIS
FCVI	Fluorescence Correction Vegetation Index: NIR−(R + G + B)/3	dimensionless	8-day	MODIS
NMBDI	Normalised Multi-Band Drought Index: (*N*−(*S*1 − *S*2))/(*N*+(*S*1 − *S*2))	dimensionless	8-day	MODIS
GLI	Green Leaf Index: (2G − R − B)/(2G + R + B)	dimensionless	8-day	MODIS
AFRI2100	Aerosol Free Vegetation Index: (NIR − 0.5 × SWIR2)/ (NIR + 0.5 × SWIR2)	dimensionless	8-day	MODIS
GVMI	Global Vegetation Moisture Index: (NIR + 0.1−(SWIR2 + 0.02))/(NIR + 0.1+(SWIR2 + 0.02))	dimensionless	8-day	MODIS
nitrogen_dioxide	Tropospheric NO_2_ column number density	mol m ^−^ ^2^	Daily	Sentinel-5P
sulphur_dioxide	SO_2_ column number density	mol m ^−^ ^2^	Daily	Sentinel-5P
carbon_monoxide	CO column number density	mol m ^−2^	Daily	Sentinel-5P
ozone	O_3_ column number density	mol m ^−^ ^2^	Daily	Sentinel-5P
elevation_m	Terrain elevation above mean sea level	m	Static	SRTM
population_density	Residential population per 100 × 100 m grid cell	persons km ^−^ ^2^	Static	WorldPop
nightlight_avg_rad	Monthly mean DNB radiance (anthropogenic activity proxy)	nW cm ^−^ ^2^ sr ^−^ ^1^	Monthly	VIIRS
sin_doy	Sine transform of day-of-year (period = 365.25 days)	dimensionless	Daily	Derived
cos_doy	Cosine transform of day-of-year (period = 365.25 days)	dimensionless	Daily	Derived
sin_dow	Sine transform of day-of-week (period = 7 days)	dimensionless	Daily	Derived
cos_dow	Cosine transform of day-of-week (period = 7 days)	dimensionless	Daily	Derived

Ground-level PM_2.5_ data were obtained from the Continuous Air Monitoring Station (CAMS) network operated by the Bangladesh Department of Environment [32]. Seven meteorological variables were retrieved from the ERA5 Reanalysis through the Open-Meteo archive [33,34]. These variables include daily mean temperature, relative humidity, precipitation, surface pressure, wind speed, wind direction, and shortwave radiation. Wind direction was averaged using a circular mean to avoid the discontinuity between 0° and 360°. Seven vegetation and surface indices were derived from the MODIS Terra MOD09A1 Surface Reflectance dataset accessed through Google Earth Engine [35]. These include NDVI, GARI, FCVI, NMBDI, AFRI2100, GLI, and GVMI. The indices represent vegetation density, canopy moisture, and aerosol sensitivity, which have known relationships with near-surface PM_2.5_ through dust loading, biomass burning smoke, and agricultural residue burning [36]. Because MOD09A1 provides 8-day composites, daily values were generated by forward-filling the most recent observation. Four atmospheric trace gas columns were obtained from Sentinel-5P TROPOMI Level-3 products: nitrogen dioxide (NO_2_), Sulphur dioxide (SO_2_), carbon monoxide (CO), and ozone (O_3_). NO_2_ and SO_2_ are key precursors of secondary aerosols, while CO indicates combustion sources such as biomass burning. O_3_ affects the atmospheric oxidation processes that control secondary particle formation [37,38]. Data gaps caused by cloud cover and orbital cycles were filled using linear interpolation. Three additional city-level variables were included. Terrain elevation was derived from the Shuttle Radar Topography Mission digital elevation model [39]. Population density was taken from the WorldPop database [40]. Monthly nighttime light intensity was obtained from the VIIRS Day/Night Band monthly composites [41] as a proxy for human activity such as traffic and industrial output. Finally, four cyclical temporal features were created from the calendar date. These include sine and cosine transformations of day-of-year and day-of-week, which allow the model to capture seasonal and weekly patterns without treating time as a linear variable.

All features were aggregated or resampled to a common daily resolution at each city location. Missing values in ground-station PM_2.5_ and meteorological variables were filled using linear interpolation with bidirectional filling to handle gaps at the beginning and end of the record, following standard practice in air quality time-series modelling [42]. Spectral indices derived from MODIS Terra were forward-filled from the most recent 8-day composite, based on the assumption that land surface conditions change slowly within such short periods. Trace gas columns from Sentinel-5P were instead linearly interpolated across missing days to avoid carrying forward values that may be outdated during extended cloud cover over Bangladesh, especially during the monsoon season. Static variables, including elevation and population density, were applied to all dates for the corresponding city. Although standard in practice, these gap-filling methods may introduce temporal bias. Linear interpolation can smooth short-lived pollution peaks, potentially underestimating brief excursions during data gaps. Forward-filling the 8-day MODIS composites assumes static land-surface conditions within each interval, an assumption that may be violated during rapid changes such as post-harvest residue burning, when indices such as GLI can vary substantially within days.

After gap filling, all input features were standardized using a StandardScaler fitted only on the training data and then applied unchanged to the validation and test sets. The final dataset contains 1,208 daily observations per city covering 2022–2025. The data were divided into training (70%), validation (15%), and test (15%) sets using sequential time-based splitting without shuffling in order to preserve temporal structure, giving 845 training days, 181 validation days, and 182 test days. Using a sliding window with a lookback of 21 days and a forecast horizon of three days produces 822 training windows, 158 validation windows, and 159 test windows.

### Spatiotemporal graph construction

The spatial relationships among the 12 study cities are encoded as a weighted undirected graph G = (V, E, A). The node set V = {v_1_, v_2_,..., v_12_} represents the 12 cities, the edge set E encodes spatial connectivity, and $A\;\in \;R^{N\times N}$ is the symmetric weighted adjacency matrix which governs how pollution signals spread across nodes during graph convolution. The graph construction pipeline (Fig 2b) is presented in four sequential stages: (i) great-circle distance computation, (ii) Gaussian kernel weighting, (iii) symmetric normalization, and (iv) stochastic edge dropout during training.

**Fig 2:**
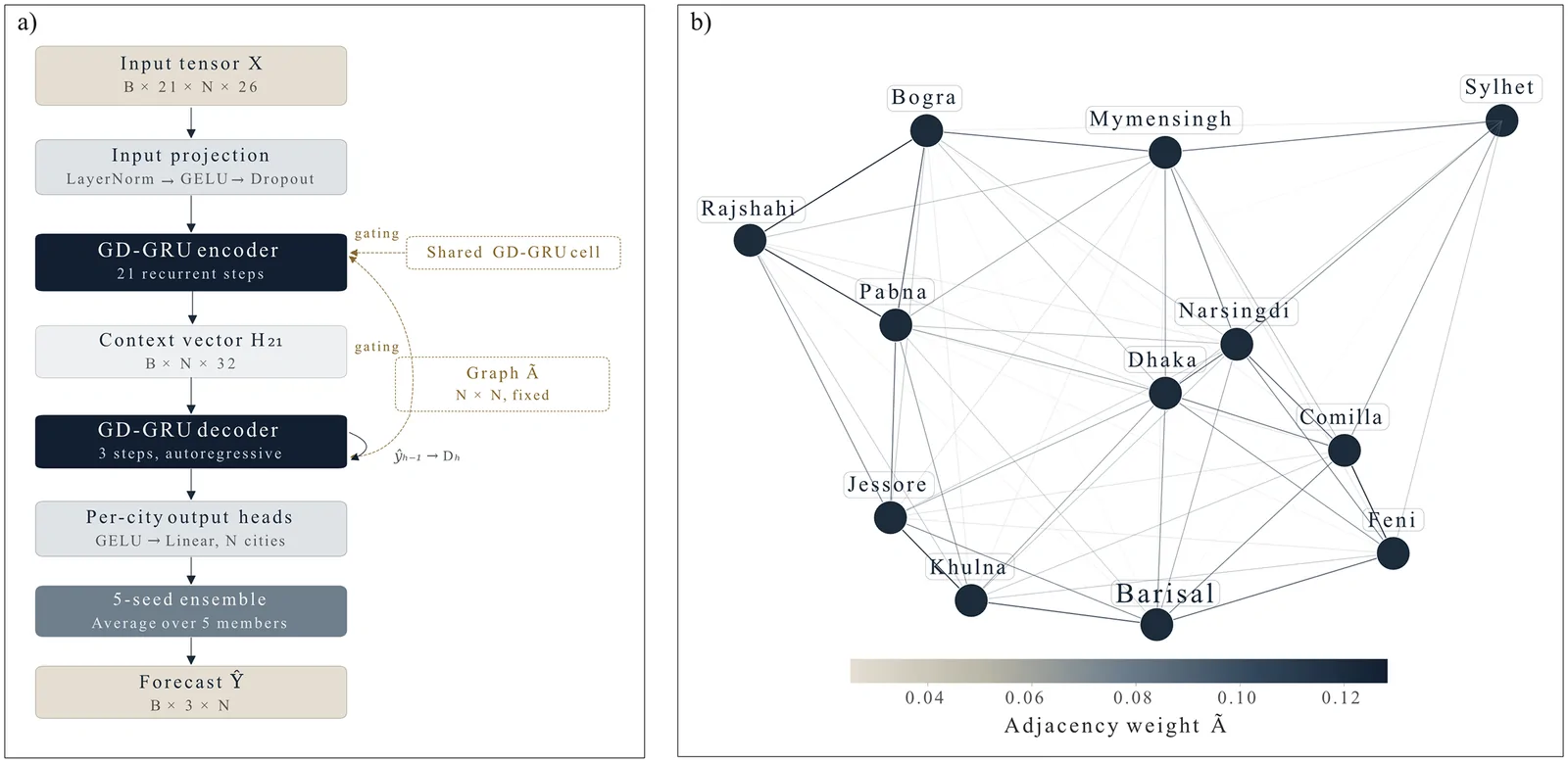
GD-GRU architecture and spatial graph. (a) Encoder-decoder pipeline showing the input projection, 21-step GD-GRU encoder, autoregressive 3-step decoder, per-city output heads, and 5-member ensemble averaging. (b) Geographic graph of the 12-city network; edge thickness reflects normalized adjacency weight in Ã.

### Great-circle distance matrix

City locations are represented by their latitude $\phi_{i}\;$and longitude $\lambda_{i}$ in radians. Pairwise geodesic distances are calculated using the Haversine formulation, which determines the shortest path between two points on a sphere of radius R=6,371 km [43,44]:


$$\begin{array}{c} a_{ij}={sin}^{\text{2}}\left(\frac{\phi_{j}-\phi_{i}}{\text{2}}\right)+\text{cos}\phi_{i}\text{cos}\phi_{j}{sin}^{\text{2}}\left(\frac{\lambda_{j}-\lambda_{i}}{\text{2}}\right) \end{array}$$ (1)

$$\begin{array}{c} d_{ij}=\text{2}R\text{arcsin}\left(\sqrt{a_{ij}}\right) \end{array}$$ (2)

The Haversine formulation is preferred over planar Euclidean distance because it provides stable great-circle distances on a spherical Earth approximation [43]. Although more precise ellipsoidal solutions exist, the spherical assumption is widely adopted in environmental and atmospheric modeling [44].

The resulting symmetric distance matrix $D\in R^{N\times N}$satisfies $d_{ii}=\text{0}\;$and $d_{ij}=d_{ji}>\text{0}\;$for i≠j. The matrix is computed once before model training and remains fixed throughout all experiments.

### Gaussian kernel edge weighting

Raw geographic distances are transformed into proximity weights using an isotropic Gaussian radial basis (RBF) kernel, a standard approach for constructing weighted graphs from pairwise distances in manifold learning and graph signal processing [45,46]. Closer cities are assigned larger weights, consistent with the physical intuition that near-field pollution transport, driven by advection, local wind circulation, and atmospheric boundary-layer mixing, is stronger between geographically proximate urban centers than between distant ones [47–49]. For i≠j:


$$\begin{array}{c} W_{ij}=\text{exp}\left(-\frac{d_{ij}^{\text{2}}}{\text{2}\sigma^{\text{2}}}\right) \end{array}$$ (3)

Self-connections are enforced as:


$$\begin{array}{c} W_{ii}=\text{1}.\text{0},\forall i \end{array}$$ (4)

The bandwidth parameter σ controls the spatial decay rate and is set to the median of all non-zero pairwise distances in D:


$$\begin{array}{c} \sigma =\text{median}\left\{d_{ij}:i\text{≠}j\right\} \end{array}$$ (5)

This choice of σ is purely data-driven and independent of scale as it adjusts automatically to the geographic spread of the city set which prevents the graph from becoming either too sparse or too dense. After applying the kernel, the diagonal is set to 1 so that each node preserves its own information during graph convolution. The resulting matrix $W\in R^{N\times N}$ is symmetric, and has all entries in the interval (0,1].

### Symmetric normalization

After edge weighting, the matrix W is symmetrically normalized. Symmetric normalization preserves the spectral properties of the graph Laplacian that guarantees the theoretical convergence of Chebyshev polynomial approximations used in spectral graph convolution [50,51]. The normalized adjacency matrix is computed as:


$$\begin{array}{c} \tilde{A}=\Delta^{-\frac{\text{1}}{\text{2}}}\;W\Delta^{-\frac{\text{1}}{\text{2}}} \end{array}$$ (6)

where $\Delta \in R^{N\times N}$is the diagonal degree matrix with entries:


$$\begin{array}{c} \Delta_{ii}=\sum_{j=\text{1}}^{N}W_{ij} \end{array}$$ (7)

and $\Delta^{-\text{1}/\text{2}}$ is computed entry-wise on the diagonal with a numerical stability floor of ${\text{1}\text{0}}^{-\text{6}}\;$to avoid division by zero. This yields the matrix $\tilde{A}\in R^{N\times N}$, which is symmetric with entries bounded in [0 ,1] under positive edge weights and has spectral radius bounded by unity.

### Stochastic edge dropout during training

To regularize the spatial pathway and reduce sensitivity to potential error in the fixed geographic adjacency, stochastic edge dropout is applied at each forward pass [52]. Each entry of $\tilde{A\;}$is independently masked with probability p=0.10:


$$\begin{array}{c} {\tilde{A}}_{ij}^{\left(\text{drop}\right)}={\tilde{A}}_{ij}\cdot \text{1}\left\{u_{ij}>p\right\},\;u_{ij}\sim \text{Uniform }(\text{0},\text{1}) \end{array}$$ (8)

The masked matrix is then row-renormalized to preserve the scale of signals:


$$\begin{array}{c} {\hat{A}}_{ij}=\frac{{\tilde{A}}_{ij}^{\left(\text{drop}\right)}}{\sum_{k=\text{1}}^{N}{\tilde{A}}_{ik}^{\left(\text{drop}\right)}+\varepsilon },\varepsilon ={\text{1}\text{0}}^{-\text{6}} \end{array}$$ (9)

Edge dropout is disabled during inference, where the full normalized $\tilde{A\;}$is used.

The resulting normalized adjacency matrix Ã is shown in Fig 3. Off-diagonal weights range from 0.02 for distant city pairs to 0.13 for the closest pairs, particularly Bogra–Rajshahi (0.13) and Feni–Comilla (0.13), reflecting the strong distance-based weighting of the Gaussian kernel. Diagonal self-weights range from 0.13 (Barisal) to 0.19 (Sylhet). Sylhet has the highest self-weight because it is relatively isolated from the main network in the northeast. Dhaka shows relatively uniform connections to all cities, with weights between 0.07 and 0.11, which is consistent with its central location in the 12-city network. The chosen bandwidth σ produces a balanced distribution of weights. It avoids values that are too small or too similar across all pairs, which helps maintain stable gradient flow during training and prevents any single city from dominating the graph convolution.

**Fig 3:**
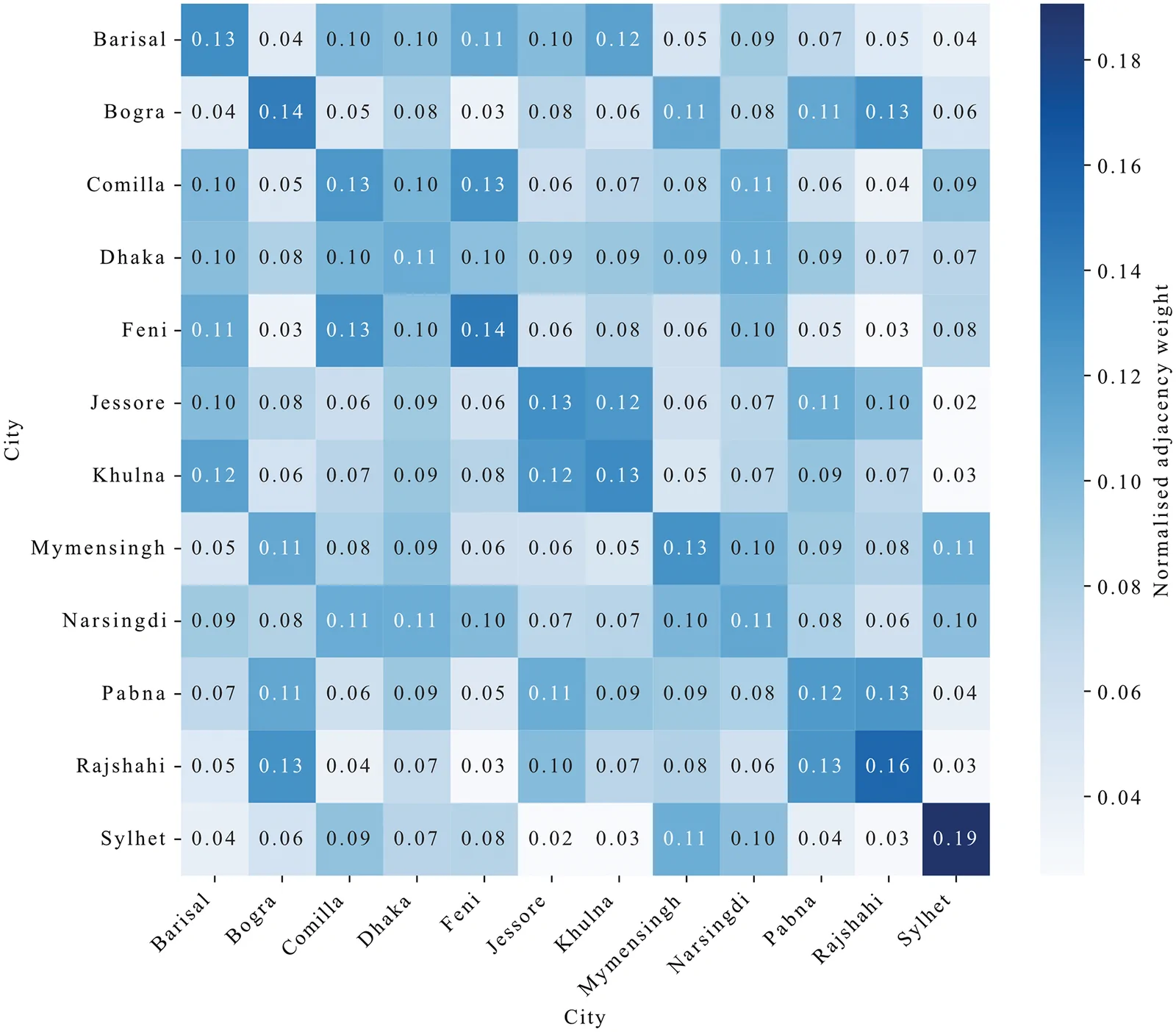
Normalized geographic adjacency matrix constructed from distance-based Gaussian kernel weighting. The matrix is built from Haversine pairwise distances and Gaussian kernel weighting, and used as the fixed graph structure throughout all experiments.

### Model architecture

Rather than proposing a fundamentally new architecture, this study implements a Chebyshev graph diffusion GRU design as an experimentally controlled framework for evaluating geographic graph validity and explainability in a data-limited setting. While convolutional, multilayer-perceptron, and generative-adversarial models excel at spatiotemporal reconstruction in other domains, such as mapping turbulent flows from sparse sensors [53,54], they typically require denser, regularly structured sensor coverage to infer full spatial fields. For Bangladesh’s sparse and irregularly spaced 12-city network, the primary challenge is capturing sequential temporal evolution and localized spatial diffusion rather than interpolating unobserved regions. Consequently, a recurrent GRU framework is better suited to this task, and its symmetric normalized adjacency formulation is theoretically consistent with the fixed geographic graph.

The GD-GRU (Graph Diffusion GRU) is an encoder-decoder spatiotemporal graph neural network architecture (Fig 2a). It replaces the standard linear projections in the gating mechanisms of a traditional Gated Recurrent Unit (GRU) with graph diffusion convolution operators. This enables each node’s hidden state to be updated based on both its own historical temporal information and the diffusion-weighted states of neighboring nodes in the graph [14,55]. The architecture has four components: an input projection module, a graph diffusion GRU encoder, an autoregressive decoder, and per-city output heads.

### Graph diffusion convolution operator

The core component of the GD-GRU is the Graph Diffusion Convolution (GDConv) layer [14], which approximates spectral graph convolution using a truncated Chebyshev polynomial expansion of order K [51]. Given input node features $\text{X}\in R^{B\times N\times d_{\text{in}\;}}\;$and the symmetrically normalized adjacency matrix $\tilde{\text{A}}\in R^{N\times N}$, the layer constructs K basis feature matrices recursively. The basis matrices are computed using the Chebyshev polynomial recurrence:


$$\begin{array}{c} T_{\text{0}}(\tilde{A})=I \end{array}$$

$$\begin{array}{c} T_{\text{1}}(\tilde{A})=\tilde{A} \end{array}$$

$$\begin{array}{c} T_{k}\left(\tilde{A}\right)=\text{2}\tilde{A}T_{k-\text{1}}\left(\tilde{A}\right)-T_{k-\text{2}}\left(\tilde{A}\right),\;k\geq \text{2} \end{array}$$ (10)

where ${\tilde{X}}_{k}={\text{T}}_{k}(\tilde{A})X$ denotes the k-th order diffused feature matrix. These are then concatenated into a linear projection:


$$\begin{array}{c} \text{GDConv}\left(\text{X},\tilde{\text{A}}\right)=\left[{\tilde{\text{X}}}_{\text{0}}\;∥\;{\tilde{\text{X}}}_{\text{1}}\;∥\;\cdots \;∥\;{\tilde{\text{X}}}_{K-\text{1}}\right]\text{W}+\text{b} \end{array}$$ (11)

where $\text{W}\in R^{(K\cdot d_{\text{in}})\times d_{\text{out}}}$ is the learnable weight matrix, and $\text{b}\in R^{d_{\text{out}}}$ is the learnable bias vector. In this study, we adopt K=2, which retains only the identity (zeroth-order) and first-order diffusion terms: X and $\tilde{\text{A}}\text{X}$. The parameter count of each GDConv layer is $K\cdot d_{\text{in}}\times d_{\text{out}}$, which is independent of the number of nodes N and remains efficient even for large graphs. Stochastic edge dropout is applied to Ã before polynomial recursion.

### Graph diffusion GRU Cell

The GD-GRU Cell replaces all three linear transformations of the standard Gated Recurrent Unit (GRU) [56] with Graph Diffusion Convolution (GDConv) operators [14]. Given input $X_{t}$ and hidden state$H_{t-\text{1}}:$


$$\begin{array}{c} r_{t}=\sigma \left({GDConv}_{r}\left([X_{t}\;||\;H_{t-\text{1}}],\tilde{A}\right)\right) \end{array}$$ (12)

$$\begin{array}{c} u_{t}=\sigma \left({GDConv}_{u}\left([X_{t}\;||\;H_{t-\text{1}}],\tilde{A}\right)\right) \end{array}$$ (13)

$$\begin{array}{c} c_{t}=tanh\left({GDConv}_{c}\left([X_{t}\;||\;r_{t}⊙H_{t-\text{1}}],\tilde{A}\right)\right) \end{array}$$ (14)

$$\begin{array}{c} {\text{H}}_{t}={\text{u}}_{t}⊙{\text{H}}_{t-\text{1}}+\left(\text{1}-{\text{u}}_{t}\right)⊙{\text{c}}_{t} \end{array}$$ (15)

Note that our update convention follows the variant used in DCRNN [14], where ${\text{u}}_{\text{t}}$ gates the previous state rather than the candidate. This is equivalent to the original GRU formulation [56] with ${\text{u}}_{\text{t}}$ replaced by $\text{1}-{\text{u}}_{\text{t}}$.

Because gating is computed through GDConv, the reset and update operations at city *i* are influenced by the recent states of geographically proximate cities. Each gate has its own independent weight matrices and biases, as denoted by the subscripts r, u, and c in $GDConv_{r}$, $GDConv_{u}$, and $GDConv_{c}$ respectively. The hidden state $H_{t}\in R^{B\times N\times H}$ captures the joint spatiotemporal representation across all cities, with H=32 in all experiments.

### Encoder

The encoder maps the input lookback window $\text{X}\in R^{B\times \text{2}\text{1}\times \text{2}\text{6}}$ (batch size B, lookback horizon $T_{\text{in}}=\text{2}\text{1}$, input feature dimension F=26) to a fixed-length context vector ${\text{H}}_{L}\in R^{B\times N\times \text{3}\text{2}}$.

At each time step t, the raw input slice ${\text{X}}_{t}\in R^{B\times N\times \text{2}\text{6}}$ is first projected to a higher-dimensional representation:


$$\begin{array}{c} \begin{array}{c} {\hat{\text{X}}}_{t}=\text{Dropout}\left(\text{GELU}\left(\text{LayerNorm}\left({\text{X}}_{t}{\text{W}}_{\text{in}}+{\text{b}}_{\text{in}}\right)\right)\right) \end{array} \end{array}$$ (16)

where, ${\text{W}}_{\text{in}}\in R^{\text{2}\text{6}\times \text{3}\text{2}}$ and ${\text{b}}_{\text{in}}\in R^{\text{3}\text{2}}$ are learnable projection parameters. LayerNorm [57] is applied independently per-city (node) and per batch element to stabilize activations across heterogeneous feature scales GELU [58] is used as the activation function. Dropout (rate 0.35; [59]) is applied after the activation for regularization.

The projected features ${\hat{\text{X}}}_{t}$ are then fed recurrently into the GD-GRU cell, initialized with zero hidden state ${\text{H}}_{\text{0}}=\text{0}$:


$$\begin{array}{c} {\text{H}}_{t}=\text{GDGRUCell}\left({\hat{\text{X}}}_{t},{\text{H}}_{t-\text{1}},\tilde{\text{A}}\right),t=\text{1},\ldots ,\text{2}\text{1} \end{array}$$ (17)

The final hidden state ${\text{H}}_{\text{2}\text{1}}\in R^{B\times N\times \text{3}\text{2}}$ serves as the sole input to the decoder.

### Autoregressive decoder

The decoder autoregressively generates the next three forecast steps ($T_{\text{out}}=\text{3}$) using a separate GD-GRU cell that shares the same architectural design as the encoder’s cell but uses independent learnable weights [60]. At each decoding step h=1,2,3, the hidden state is updated as follows:


$$\begin{array}{c} {\text{H}}_{h}={\text{GDGRUCell}}_{\text{dec}}\left({\text{D}}_{h},{\text{H}}_{h-\text{1}},\tilde{\text{A}}\right) \end{array}$$ (18)

Here, H_0_ = H_21_ is the encoder context vector that initializes the decoder. The decoder input $D_{h}\;\in \;R^{\left\{B\times N\times \text{1}\right\}}$ carries a single PM_2.5_ value per-city at each step: at h = 1, this is the last observed value from the lookback window; at h = 2 and h = 3, it is the model’s own prediction from the previous step. During training, teacher forcing [61] is applied with a linearly decaying schedule to stabilize gradient flow during early training and progressively reduce the gap between training and inference conditions:


$$\begin{array}{c} \rho_{\text{epoch}}=\text{max}\left(\text{0},\;\text{0}.\text{6}\times \left(\text{1}-\frac{\text{epoch}}{\text{3}\text{0}\text{0}}\right)\right) \end{array}$$ (19)

The ratio begins at $\rho_{\text{0}}=\text{0}.\text{6}$ and decays linearly toward zero over the full 300-epoch maximum training budget. At inference, teacher forcing is disabled (ρ=0).

### Per-city output projection heads

Following each decoder step, the hidden state ${\text{H}}_{h}\in R^{B\times N\times \text{3}\text{2}}$ is passed through a two-stage per-city output projection to produce the forecast for the target variable. The projection proceeds as follows:


$$\begin{array}{c} {\text{S}}_{h}=\text{Dropout}\left(\text{GELU}\left({\text{H}}_{h}{\text{W}}_{s}+{\text{b}}_{s}\right)\right) \end{array}$$ (20)

$$\begin{array}{c} {\hat{\text{O}}}_{h}={\text{S}}_{h}{\text{W}}_{c}+{\text{b}}_{c},{\text{W}}_{c}\in R^{H\times N},{\text{b}}_{c}\in R^{N} \end{array}$$ (21)

Here $S_{h}\in R^{B\times N\times H}$, so each city’s H-dimensional representation is projected to an N-dimensional vector, yielding ${\hat{\text{O}}}_{h}\in R^{B\times N\times N}$. The forecast for city i at horizon h is then extracted from the diagonal:


$$\begin{array}{c} {\hat{y}}_{h,i}={\hat{\text{O}}}_{h}[.,i,i] \end{array}$$ (22)

This design assigns each city a dedicated output neuron within the shared projection. This allows the model to learn city-specific transformations from the common hidden representation. The final predictions across all three forecast horizons are collected as:


$$\begin{array}{c} \hat{\text{Y}}=\text{stack}\left({\hat{\text{y}}}_{\text{1}},{\hat{\text{y}}}_{\text{2}},{\hat{\text{y}}}_{\text{3}}\right)\in R^{B\times \text{3}\times N} \end{array}$$ (23)

### Ensemble strategy

To improve prediction robustness and reduce model variance, five independent GD-GRU instances are trained using different random seeds: {42, 7, 123, 999, 2024}. These seeds influence weight initialization, batch ordering, dropout masks, and any stochastic noise realizations during training. The final ensemble prediction is obtained by simple arithmetic averaging across the five models:


$$\begin{array}{c} \hat{\text{Y}}=\frac{\text{1}}{\text{5}}\sum_{k=\text{1}}^{\text{5}}{\hat{\text{Y}}}^{\left(k\right)} \end{array}$$ (24)

where ${\hat{\text{Y}}}^{\left(k\right)}\in R^{B\times \text{3}\times N}$ denotes the forecast tensor produced by the k-th GD-GRU instance (with identical architecture and hyperparameters but different random initialization and stochastic behavior).

### Training strategy

The model is trained using four main components: a weighted loss function, a multi-mechanism regularization stack, an optimization schedule, and an early stopping criterion. All five ensemble members share identical hyperparameters, with stochasticity from differences in random seeds.

### Loss function

We use a weighted Huber loss as the training objective to address the strongly right-skewed distribution of PM_2.5_ concentrations in Bangladesh. Unlike mean squared error, Huber loss avoids gradient instability caused by outliers [62]. The Huber loss is defined as:


$$\begin{array}{c} L_{\delta }(z)=\left\{ \begin{array}{cc} \frac{\text{1}}{\text{2}}z^{\text{2}} & |z|\leq \delta \\ \delta \left(|z|-\frac{\delta }{\text{2}}\right) & |z|>\delta \end{array}\right. \end{array}$$ (25)

where $z={\hat{y}}_{h,i}-y_{h,i}$ is the prediction error for horizon h and city i, and the transition parameter is fixed at δ=1.0. We apply horizon weights to the loss, giving higher importance to shorter forecast horizons, since short-term air quality predictions are usually more useful in practice. We calculate these weights once using the validation set and keep them fixed throughout all experiments. The adopted per-horizon weights are 1.4 for +1-day, 1.2 for +2-day, and 1.0 for +3-day.

### Regularization

To reduce overfitting on the limited training data (approximately 822 windows), four complementary regularization strategies are applied simultaneously. Stochastic edge dropout (p = 0.10) on the normalized adjacency matrix Ã is applied at every forward pass. Standard dropout (rate = 0.35) is applied after the input projection layer and after every GD-GRU cell output in both the encoder and decoder [59] adding independent noise at each time step. L2 weight decay (λ = 5 × 10 ^−^ ³) is imposed on all trainable parameters through the AdamW optimizer [63]. Finally, Gaussian input noise (σ = 0.15) is added to the standardized input during training. This helps the model handle measurement noise that exists in real monitoring data [64].


$$\begin{array}{c} {\text{X}}^{\prime }=\text{X}+\epsilon ,\epsilon \sim \text{N}\left(\text{0},\sigma^{\text{2}}\text{I}\right) \end{array}$$ (26)

### Optimisation

We optimize the parameters using AdamW [63] with the following settings: Initial learning rate: $\text{3}\times {\text{1}\text{0}}^{-\text{4}}$, Gradient clipping: maximum norm of 3.0, Batch size: 32. We reduce the learning rate by a factor of 0.5 whenever the validation loss stops improving for 15 epochs, with a minimum learning rate floor of $\text{5}\times {\text{1}\text{0}}^{-\text{7}}$.

### Early stopping

We stop training when the smoothed validation loss does not decrease by more than ${\text{1}\text{0}}^{-\text{4}}$ for 40 consecutive epochs. We enforce a minimum of 80 warmup epochs before early stopping can take effect. The smoothed validation loss is calculated using an exponential moving average with smoothing coefficient α=0.3 (weight on the historical average):


$$\begin{array}{c} L_{e}^{\text{smooth}}=\text{0}.\text{3}\cdot L_{e-\text{1}}^{\text{smooth}}+\text{0}.\text{7}\cdot L_{e}^{\text{val}} \end{array}$$ (27)

This smoothing helps avoid stopping too early during the teacher-forcing decay phase, when validation loss can fluctuate. At the end of training, we restore the checkpoint that achieved the lowest smoothed validation loss. We summarize all training hyperparameters in Table 2.

**Table 2:** GD-GRU model hyperparameters.

Parameter	Value
**Optimiser**	AdamW
**Learning rate**	3 × 10 ^−^ ^4^
**Weight decay**	5 × 10 ^−^ ^3^
**Gradient clip norm**	3.0
**LR reduction factor/ patience**	0.5/ 15 epochs
**Batch size**	32
**Max epochs**	300
**Warmup epochs**	80
**Early stop patience**	40 epochs
**EMA smoothing**	(α) → 0.3
**Huber δ**	1.0
**Input noise σ**	0.15
**Dropout rate**	0.35
**DropEdge rate**	0.10
**Teacher forcing (initial)**	0.6
**Horizon loss weights**	+1d: 1.4, + 2d: 1.2, + 3d: 1.0
**Ensemble size**	5 seeds

### Explainability analysis

To interpret how and why the GD-GRU produces its forecasts, we apply five complementary explainability methods to the trained ensemble on the test set. Because any single attribution technique may introduce method-specific artifacts, we consider consistent rankings across independent methods as robust evidence of genuine feature or city importance [65].

### Gradient × Input

We compute saliency maps by backpropagating gradients from the forecast output to the input tensor and performing an element-wise multiplication with the input magnitude [66,67]:


$$\begin{array}{c} \phi_{f}^{\left(h\right)}=E_{s}\left[|\frac{\partial {\hat{y}}^{\left(h\right)}}{\partial x_{f}}⊙x_{f}|\right] \end{array}$$ (28)

where the expectation is taken over $N_{s}=\text{2}\text{0}\text{0}$ randomly sampled test windows. This gives a feature importance score $\phi_{f}^{\left(h\right)}$ for each input feature f and forecast horizon h. We report both horizon-averaged scores and per-horizon breakdowns to see whether the model’s reliance on different features changes with lead time.

### Temporal sensitivity

To quantify the contribution of each lookback timestep to each forecast horizon, we compute the gradient norm aggregated across all features [68]:


$$\begin{array}{c} \tau_{t}^{\left(h\right)}=E_{s}\left[{∥\frac{\partial {\hat{y}}^{\left(h\right)}}{\partial {\text{X}}_{t}}∥}_{F}\right],t=\text{1},\ldots ,\text{21} \end{array}$$ (29)

where $∥\cdot {∥}_{F}$ denotes the Frobenius norm over cities and features. A high value of $\tau_{t}^{\left(h\right)}$ indicates that the model is especially sensitive to conditions observed 21-t days before the forecast. This reveals the effective memory length the encoder uses for each prediction horizon.

### Permutation feature importance

We estimate each feature’s contribution using permutation feature importance (PFI). Specifically, we measure the increase in test MAE when a given feature is randomly permuted across samples, thereby disrupting its association with the target while preserving its marginal distribution [69]:


$$\begin{array}{c} {\text{PFI}}_{f}=\frac{\text{1}}{R}\sum_{r=\text{1}}^{R}\left[\text{MAE}\left({\hat{Y}}^{\left(f,r\right)}\right)-\text{MAE}\left(\hat{Y}\right)\right] \end{array}$$ (30)

where R=5 repeats are used to estimate variance. ${\hat{Y}}^{\left(f,r\right)}$ is the ensemble prediction after the r-th permutation. Unlike gradient methods, permutation importance does not require differentiability and captures nonlinear interactions. We report the mean and standard deviation across the five repeats.

### SHAP values

We apply KernelSHAP [26] to estimate the marginal contribution of each feature to the + 1-day forecast. The model is wrapped as a function of the temporally averaged input, collapsing the 21-day lookback window into a single feature vector per sample. KernelSHAP treats the model as a black box; thus, spatial relations in the graph are reflected only indirectly through their influence on the final output. This is a known limitation when applying additive attribution methods to graph-based models [70]. Furthermore, collapsing the 21-day lookback window into a single averaged feature vector restricts KernelSHAP’s ability to resolve time-lag effects, such as the delayed influence of wind speed from preceding days. Permutation feature importance similarly disrupts the temporal ordering of the lookback window when a feature is shuffled across samples. We treat this as a current constraint of applying additive, tabular-style attribution methods to spatiotemporal graph models rather than a property of the underlying feature effects.

### Spatial influence

We quantify city-level spatial influence through an occlusion analysis [71]. For each source city i, we mask its entire feature sequence to zero and measure the resulting change in the + 1d forecast across all target cities j:


$$\begin{array}{c} \Omega_{ij}=E_{s}\left[|{\hat{y}}_{j}^{\;\text{masked}(i)}-{\hat{y}}_{j}|\right],N_{s}=\text{200} \end{array}$$ (31)

The resulting matrix $\Omega \in R^{N\times N}$ is a spatial influence map that we compare directly against the fixed adjacency $\tilde{A}$. We use Pearson correlation between the off-diagonal elements of Ω and $\tilde{A}$ as a graph validation metric. A high correlation indicates that the geographic adjacency is a reliable proxy for the spatial influence structure learned by the model.

To quantify agreement across the three feature-attribution methods beyond visual comparison of importance rankings, the Spearman rank correlation and the top-10 feature-set Jaccard overlap are computed between each pair of methods (Gradient × Input, PFI, KernelSHAP) over their shared feature set.

### Baseline models

We evaluate the model against six baseline models. The models represent three levels of complexity. This evaluation strategy is designed to identify the contribution of temporal modeling, spatial structure, and deep learning capacity.

**Persistence**: It predicts the last observed PM_2.5_ value as a flat forecast across all horizons:


$$\begin{array}{c} {\hat{y}}_{t+h}=y_{t},h=\text{1},\text{2},\text{3} \end{array}$$ (32)

This simple method sets the baseline for basic forecasting performance.

**Climatological Mean:** It forecasts the average historical PM_2.5_ for the target day of the year, using a ± 7-day rolling window from training data. This checks if models rely on seasonal patterns instead of true day-to-day predictability and measures how much variance comes from seasonality alone.

**Random Forest:** As a strong non-recurrent baseline, we implement a Random Forest Regressor to predict daily ${PM}_{\text{2}.\text{5}}$ concentrations at each city for the three forecast horizons (h=1,2,3) [72]. We flatten the spatiotemporal input into a tabular format. For each sample, we use the full 21-day lookback window of the 26 input features. RF serves as a strong point of comparison because its ensemble bagging approach effectively captures nonlinear relationships among meteorological and temporal variables while remaining highly resistant to overfitting.

**XGBoost:** It follows the same per-city per-horizon structure as RF using gradient-boosted trees [73]. XGBoost fundamentally differs from RF by optimizing predictions sequentially; it iteratively builds shallow decision trees that specifically target and correct the residual errors of prior trees. This baseline is included because its internal $L_{\text{1}}$ and $L_{\text{2}}$ regularization mechanisms, combined with its efficient handling of complex feature interactions, frequently yield state-of-the-art performance on tabularized environmental datasets, providing a rigorous empirical benchmark against the deep spatiotemporal graph models.

**GRU (No Graph):** To isolate the spatial graph component, we include a GRU (no graph) ablation as a direct counterpart to the full GD-GRU model. This variant uses a five-seed ensemble with an identical architecture to GD-GRU, except all Graph Diffusion Convolution (GDConv) operators are replaced by standard dense linear projections. This eliminates graph-based message passing and spatial diffusion, reducing the model to a classic recurrent sequence processor that handles each city’s time series independently.

**DCRNN:** The Diffusion Convolutional Recurrent Neural Network replaces GRU linear transformations with bidirectional random-walk diffusion convolution operators [14]. Forward and backward transition matrices $T_{f}=D_{out}^{-\text{1}}A$ and $T_{b}=D_{in}^{-\text{1}}A^{⊤}$are computed from the same geographic adjacency used by GD-GRU, ensuring a controlled comparison. The model uses K=2 diffusion steps, hidden dimension 32, and an identical 5-seed ensemble, training pipeline, loss function, and regularization stack to GD-GRU. The only architectural difference is the diffusion operator: bidirectional random-walk (DCRNN) versus Chebyshev polynomial approximation (GD-GRU).

We report all hyperparameters for the baselines in Table 3. The GRU (no graph) ensemble and DCRNN follow the same training pipeline as the full GD-GRU model.

**Table 3:** Baseline model hyperparameters.

Model	Configuration
**Persistence**	None (forecast(t + h) = observed(t))
**Climatology**	±7-day DOY rolling window, training set only. Boundary handling: the window indexes with ((doy + d − 1) mod 365) + 1, i.e., it wraps circularly — Dec 29–31 borrow from early January and Jan 1–3 borrow from late December, with no truncated or asymmetric window at the year boundary.
**Random Forest**	n_estimators = 300, max_depth = 12, min_samples_split = 5, min_samples_leaf = 2, max_features = “sqrt”, input: flattened [21 × F]
**XGBoost**	n_estimators = 300, max_depth = 6, lr = 0.05, subsample = 0.8, colsample_bytree = 0.8, gamma = 0.1, reg_alpha = 0.1, reg_lambda = 1.0
**GRU (no graph)**	hidden = 32, dropout = 0.35, 5 seeds. All GDConv operators are replaced (both encoder and decoder, all three gates) — the encoder GDGRUCell → standard GRU; the decoder GDGRUCell → standard GRUCell.
**DCRNN**	hidden = 32, K = 2, bidirectional diffusion, 5 seeds; same training pipeline as GD-GRU

### Statistical significance testing

To assess whether performance differences between GD-GRU and each baseline are statistically meaningful, we apply two complementary tests. The Diebold-Mariano (DM) test [74] with Harvey-Leybourne-Newbold small-sample correction [75] evaluates equal predictive accuracy under squared error loss. The null hypothesis is that two forecasts have equal expected loss; the alternative is two-sided. The Wilcoxon signed-rank test evaluates whether the median absolute error of GD-GRU is lower than each baseline (one-sided alternative). Because the DM test is sensitive to large squared errors while the Wilcoxon test is rank-based, consistent results across both tests provide stronger evidence than either test alone. All tests are conducted at the α = 0.05 significance level.

### Implementation and computational details

We implement all deep learning models (GD-GRU, GRU (no graph), DCRNN, and related components) in PyTorch 2.0 [76]. For the tree-based baselines, we use scikit-learn 1.3 for Random Forest [77] and XGBoost 2.0 for gradient boosting [73].

All experiments are run on a single NVIDIA GPU with CUDA 11.8. Training the full five-member GD-GRU ensemble takes approximately 45 minutes on average. The GRU (no graph) ensemble is somewhat faster, requiring about 35 minutes, due to the lower computational cost per time step. The DCRNN model required the highest computation time, taking 1 hour and 15 minutes. In contrast, training each Random Forest and XGBoost configuration completes in under 15 minutes. We fix all random seeds across every source of stochasticity. This includes weight initialization, data shuffling, dropout mask generation, and input noise realizations. For each ensemble member, we select the final checkpoint based on the lowest smoothed validation loss, and the early stopping criteria.

## Results

### Overall forecasting performance

Table 4 shows overall test set performance for all six models across all cities and forecast horizons. The GD-GRU ensemble achieves competitive performance (MAE = 8.853 µg/m^3^, RMSE = 12.139 µg/m^3^, MAPE = 34.13%, R^2^ = 0.666, and PCC = 0.819). GD-GRU outperforms DCRNN across all pooled metrics, including MAE, RMSE, MAPE, R^2^, and PCC, demonstrating a consistent, albeit modest, performance advantage. The naive Persistence and Climatological Mean baselines achieve moderate R^2^ values of 0.513 and 0.562, respectively, reflecting genuine day-to-day and seasonal autocorrelation in PM_2.5_ during the test period and establishing a meaningful benchmark. GD-GRU substantially improves upon both baselines, reducing MAE by 13.8% relative to Persistence and 17.1% relative to Climatology, indicating that it captures predictive structure beyond simple autocorrelation and seasonal averaging. When evaluated against a strong machine learning baseline, Random Forest, the GD-GRU model achieved an 8.34% relative improvement in MAE, reducing the error from 9.658 µg/m^3^ to 8.853 µg/m^3^, along with an absolute R^2^ improvement of 0.0263. A similar trend was observed when comparing the model with XGBoost. While XGBoost produced a MAPE of 38.05%, GD-GRU achieved a lower MAPE of 34.13%, representing an absolute reduction of 3.92 percentage points (10.3% relative improvement). Additionally, the model demonstrated an absolute R^2^ gain of 0.032 (from 0.634 to 0.666) and an absolute PCC gain of 0.021 (from 0.797 to 0.819). The GRU (no graph) baseline achieves MAE = 9.225 µg/m^3^ and R^2^ = 0.637. The GD-GRU ensemble reduces MAE by a further 4.03% and improves R^2^ by 0.029 over this direct ablation, demonstrating a statistically interpretable contribution from the graph diffusion component.

**Table 4:** Overall test set performance.

Model	MAE	MAE CI	RMSE	MAPE (%)	R^2^	R^2^ CI	PCC
Persistence	10.265	[8.352, 12.180]	14.672	39.65	0.5127	[0.2596, 0.6320]	0.7490
Climatology	10.678	[8.590, 12.559]	13.908	42.48	0.5622	[0.3108, 0.6686]	0.7561
Random Forest	9.658	[7.950, 11.238]	12.609	40.50	0.6401	[0.4497, 0.7185]	0.8006
XGBoost	9.358	[7.758, 11.012]	12.709	38.05	0.6344	[0.4602, 0.7135]	0.7974
GRU (no graph)	9.225	[7.598, 10.788]	12.659	38.59	0.6373	[0.4642, 0.7170]	0.7991
DCRNN	8.942	[7.340, 10.591]	12.260	36.04	0.6598	[0.5116, 0.7338]	0.8127
**GD-GRU (ours)**	**8.853**	**[7.207, 10.535]**	**12.139**	**34.13**	**0.6664**	**[0.4936, 0.7449]**	**0.8187**

Statistical significance testing (Table 5) confirms that GD-GRU improvements over GRU (no graph), Random Forest, and XGBoost are statistically significant overall (DM test p < 0.001; Wilcoxon p < 0.001). GD-GRU leads DCRNN on every pooled metric in Table 4 (MAE, RMSE, MAPE, R^2^, and PCC), and this advantage reaches statistical significance when compared jointly across all forecast horizons (FDR-corrected DM test p < 0.001). At the level of individual horizons the two models remain closely matched (FDR-corrected DM test: p = 0.755, 0.147, and 0.051 for +1-day, + 2-day, and +3-day, respectively), with GD-GRU’s edge concentrated at the + 2-day and +3-day horizons (Cohen’s d = −0.041 pooled; absolute MAE difference of 0.089 µg m ^−^ ^3^, 1.0%). The significance of the graph diffusion contribution is demonstrated by the GRU (no graph) comparison, where GD-GRU achieves p < 0.001 overall.

**Table 5:** Statistical significance of GD-GRU vs. baseline models, with multiplicity correction and effect sizes.

Horizon	Baseline	MAE diff [95% CI]	DM p (FDR-BH)	Cohen’s d	Cohen’s Sig.	Wilcoxon p	Wilcoxon Sig.
**ALL**	Persistence	−1.412 [−1.759, −1.068]	<0.001	−0.195	***	<0.001	***
**+1d**	Persistence	−0.483 [−0.774, −0.213]	<0.001	−0.107	***	<0.001	***
**+2d**	Persistence	−1.415 [−1.994, −0.864]	<0.001	−0.190	***	<0.001	***
**+3d**	Persistence	−2.338 [−3.080, −1.576]	<0.001	−0.260	***	<0.001	***
**ALL**	Climatology	−1.825 [−2.212, −1.418]	<0.001	−0.261	***	<0.001	***
**+1d**	Climatology	−3.626 [−4.390, −2.886]	<0.001	−0.456	***	<0.001	***
**+2d**	Climatology	−1.300 [−1.971, −0.603]	<0.001	−0.195	***	<0.001	***
**+3d**	Climatology	−0.549 [−1.116, 0.041]	0.157	−0.094	ns	<0.001	***
**ALL**	Random Forest	−0.805 [−1.096, −0.496]	<0.001	−0.138	***	<0.001	***
**+1d**	Random Forest	−1.746 [−2.207, −1.281]	<0.001	−0.308	***	<0.001	***
**+2d**	Random Forest	−0.603 [−1.151, −0.053]	0.067	−0.104	ns	<0.001	***
**+3d**	Random Forest	−0.065 [−0.575, 0.456]	0.237	−0.011	ns	0.325	ns
**ALL**	XGBoost	−0.505 [−0.725, −0.268]	<0.001	−0.087	***	<0.001	***
**+1d**	XGBoost	−0.466 [−0.714, −0.221]	0.002	−0.117	**	<0.001	***
**+2d**	XGBoost	−0.639 [−1.065, −0.232]	<0.001	−0.108	***	<0.001	***
**+3d**	XGBoost	−0.411 [−0.924, 0.086]	0.167	−0.058	ns	0.036	*
**ALL**	GRU (no graph)	−0.372 [−0.635, −0.115]	<0.001	−0.080	***	<0.001	***
**+1d**	GRU (no graph)	−0.209 [−0.460, 0.043]	<0.001	−0.066	***	<0.001	***
**+2d**	GRU (no graph)	−0.292 [−0.750, 0.134]	0.012	−0.062	*	0.011	*
**+3d**	GRU (no graph)	−0.615 [−1.168, −0.058]	0.012	−0.107	*	<0.001	***
**ALL**	DCRNN	−0.089 [−0.209, 0.039]	<0.001	−0.041	***	0.005	**
**+1d**	DCRNN	0.012 [−0.110, 0.133]	0.755	0.009	ns	0.644	ns
**+2d**	DCRNN	−0.073 [−0.284, 0.125]	0.147	−0.034	ns	0.108	ns
**+3d**	DCRNN	−0.204 [−0.468, 0.060]	0.051	−0.074	ns	<0.001	***

a.DM: Diebold-Mariano test (HLN correction, two-sided), Benjamini–Hochberg FDR-corrected across all 24 comparisons (α = 0.05).

b.*** p < 0.001, ** p < 0.01, * p < 0.05, ns: not significant.

c.Wilcoxon: one-sided signed-rank test on |error| (raw p-values, uncorrected).

Per horizon performance analysis is presented in Table 6. The model performed best at 1 day lead time. The + 1-day forecast achieves R^2^ = 0.768 and MAE = 6.989 µg/m^3^. As expected, the performance has dropped at longer lead times. Performance at the + 2-day and +3-day horizons decreases to R^2^ = 0.644 and R^2^ = 0.594 respectively, with corresponding MAE increases to 9.347 and 10.223 µg/m^3^. The observed versus predicted scatter plots (Fig 4) illustrate this degradation visually. At +1 day, predictions align tightly along the 1:1 reference line across the full observed range (3.66–139.14 µg/m^3^), with only modest scatter at high concentrations. At longer lead times the model shows a pattern of underpredicting extreme pollution events. Importantly, the + 3-day scatter plot retains a well-defined linear structure (R^2^ = 0.594). It confirms that useful predictive information persists at the 72-hour horizon even in this data-scarce setting.

**Table 6:** GD-GRU ensemble performance by forecast horizon.

Horizon	MAE (μg/m^3^)	RMSE (μg/m^3^)	MAPE (%)	R^2^	PCC
**+1 day**	6.989	9.911	26.58	0.768	0.877
**+2 day**	9.347	12.477	36.22	0.644	0.806
**+3 day**	10.223	13.718	39.58	0.594	0.776

**Fig 4:**
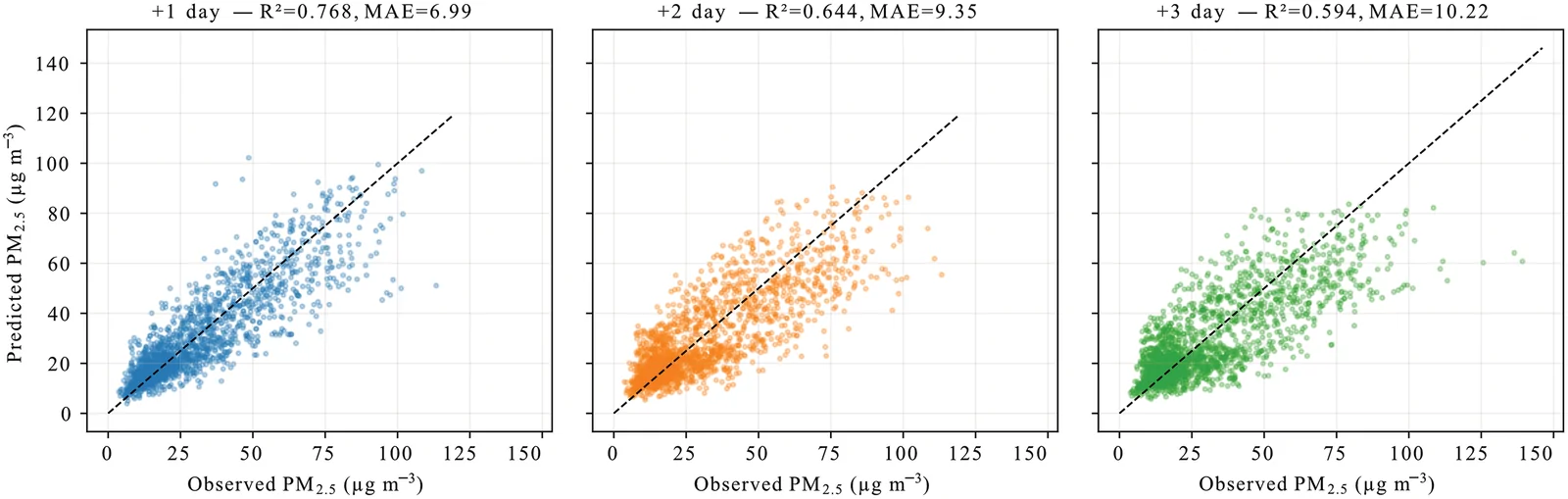
Observed versus predicted PM_2.5_ across the three forecast horizons. Scatter plots at the + 1-day (left), + 2-day (centre), and +3-day (right) horizons; dashed line indicates the 1:1 reference.

The R^2^ heatmap (Fig 5) reveals substantial heterogeneity in model performance across the 12 study cities. At the + 1-day horizon, R^2^ ranges from 0.65 (Bogra) to 0.80 (Barisal, Khulna, Narsingdi). Sylhet presents a particularly interesting case. Despite achieving one of the lowest overall R^2^ values (0.534), it records the lowest MAE of all cities at 6.520 µg/m^3^. This apparent paradox is explained by Sylhet’s characteristically low $PM_{\text{2}.\text{5}}$ variability. Its northeastern hilly terrain and relative isolation from major emission corridors keep air quality stable [31], which reduces absolute prediction errors while simultaneously limiting the variance the model needs to explain. The result is a low R^2^ despite accurate magnitude predictions. Bogra and Mymensingh show the weakest predictive performance among all cities. Bogra records the highest MAE of 11.548 µg/m^3^ alongside an R^2^ of 0.552, while Mymensingh returns an R^2^ of 0.529. Both are north-central cities where pollution patterns are driven by three main factors: transboundary airflow from the Indo-Gangetic Plain, local brick kiln emissions, and heterogeneous land use [4,5]. Barisal and Khulna achieve the strongest overall predictive performance, with R^2^ values of 0.704 and 0.709 and MAE values of 7.713 and 8.072 µg/m^3^ respectively. Both cities are located in southern Bangladesh and share similar coastal meteorological conditions. Finally, Dhaka achieves a MAE of 9.164 µg/m^3^ and an overall R^2^ of 0.655, with its best performance at the + 1-day horizon (R^2^ = 0.780).

**Fig 5:**
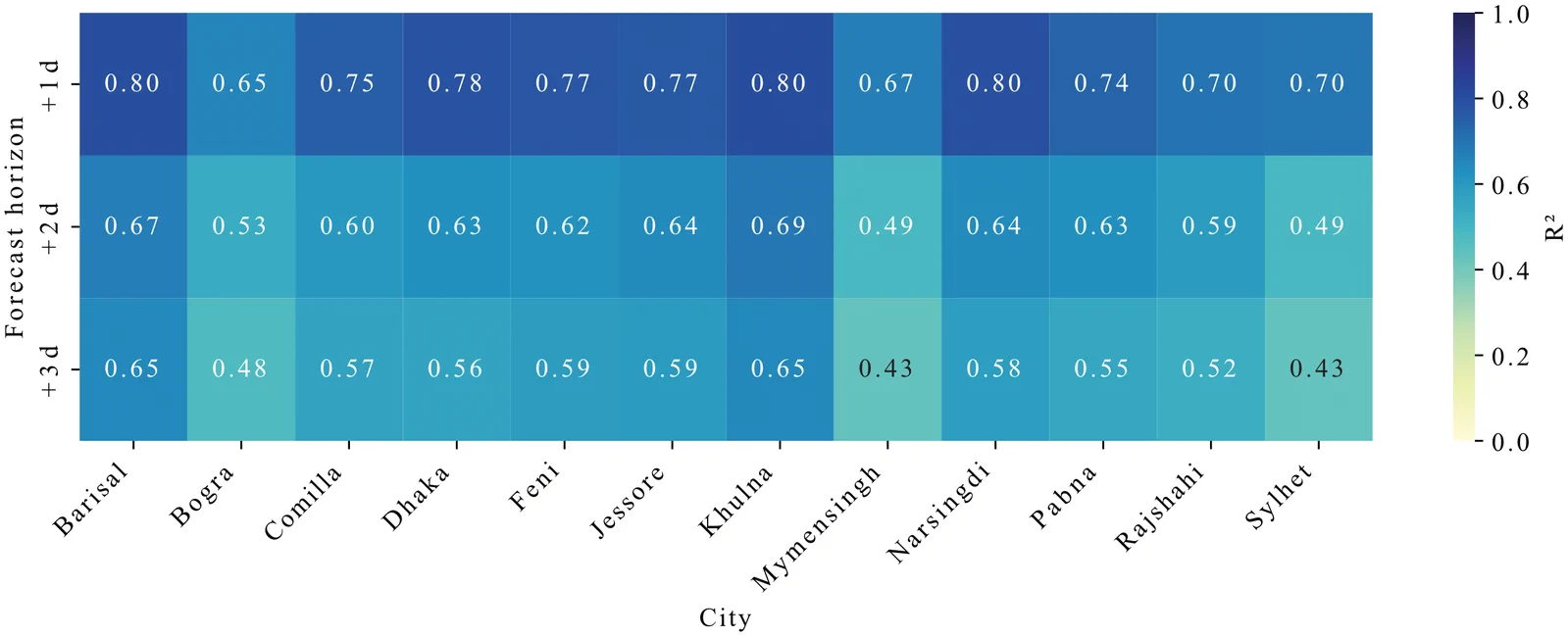
R^2^ performance heatmap across the 12 cities and three forecast horizons.

The time-series overlay (Fig 6) for representative cities supports these patterns. The model captures the seasonal transition from the cleaner monsoon period to the dry-season pollution build-up for cities such as Barisal, Dhaka, and Comilla. In these locations, the ensemble uncertainty bands (shaded area) remain relatively narrow, indicating stable predictions. For Bogra, the model captures the general seasonal pattern but fails to reproduce sudden spike-and-recovery events. This suggests that short-term local emission sources may play a stronger role in driving variability in this city.

**Fig 6:**
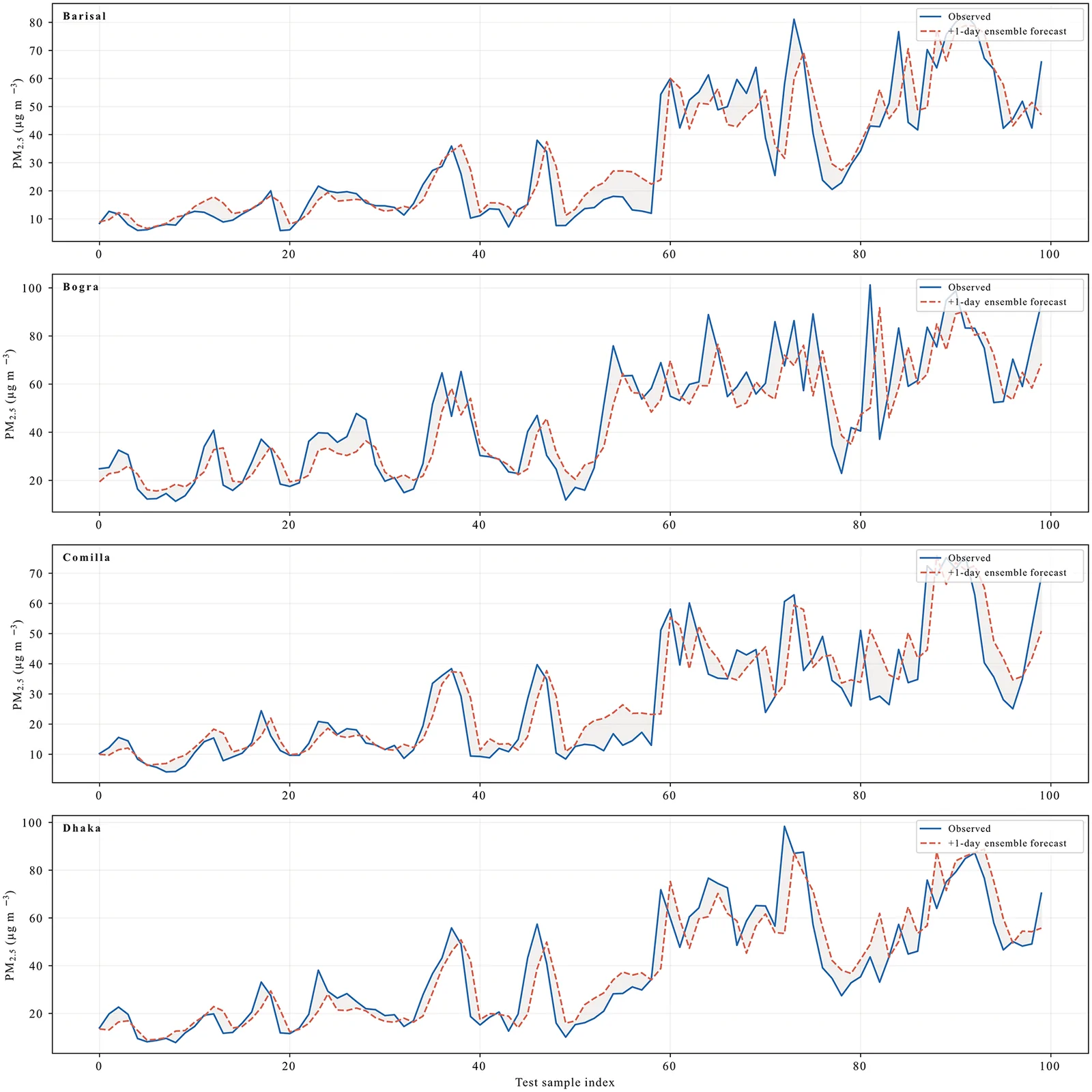
Observed and +1-day forecast PM_2.5_ time series for four representative cities. Solid lines show observed values and dashed lines show the ensemble forecast; shaded bands indicate ensemble spread.

### Training stability

The training and validation loss curves for all five ensemble members are shown in Fig 7. All models converge smoothly from initial scaled Huber loss values of about 0.45–0.46 for training and 0.69–0.71 for validation. The curves stabilize after roughly 120 epochs, with no signs of divergence, strong oscillation, or overfitting. The curves from all five random seeds remain very close to each other, despite differences in weight initialization, dropout masks, and batch ordering. This consistency indicates that the training process is stable.

**Fig 7:**
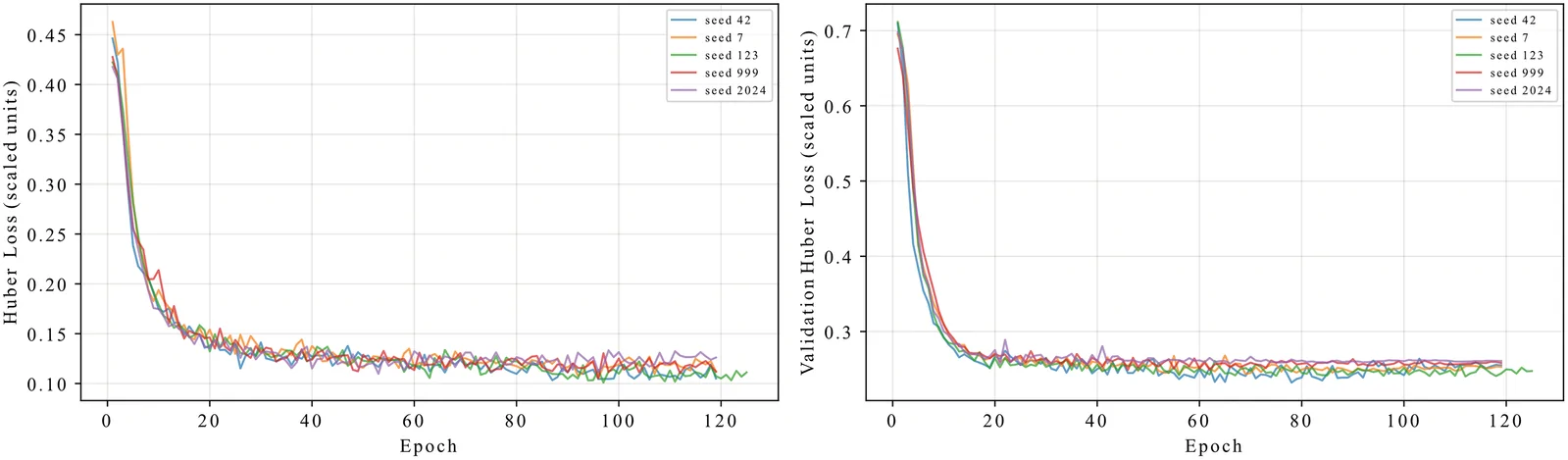
Training and validation loss curves for all five ensemble members. Huber loss across 120 epochs (training, left; validation, right), demonstrating convergence stability across random seeds.

### Explainability results

#### Convergent feature importance across three independent methods.

Table 7 presents the top-10 feature rankings produced by each explainability method. Four features, GLI, wind_speed, pressure, and cos_doy, appear within the top five in all three methods, forming a consistent core of the most influential predictors. This convergence is statistically significant: all three pairwise Spearman rank correlations are positive and significant at p<0.005, with the strongest agreement observed between Gradient × Input and SHAP (ρ=0.783, $p=\text{3}.\text{7}\times {\text{1}\text{0}}^{-\text{6}}$) and the weakest between Gradient × Input and PFI (ρ=0.590, p=0.0019). Top-10 feature-set overlap ranges from 0.538 to 0.667 across method pairs (S1 Table), confirming a robust consensus on the primary predictive drivers while capturing the complementary analytical strengths of each technique across global seasonal dependencies and localized sample-level sensitivities. As a clear demonstration of this agreement, GLI (Green Leaf Index) exhibits dominant importance across all methods, ranking first in Gradient × Input (Fig 8a) and SHAP (Fig 8c), and second in PFI (Fig. 8b), consistently outpacing NDVI (ranked 9th, 10th, and 5th, respectively).

**Table 7:** Feature importance convergence across three independent XAI methods, with raw scores and [0,1] min–max normalized scores shown together.

Rank	Gradient × Input (raw/ norm)	PFI, MAE Δ µg/m³ (raw/ norm)	SHAP, + 1d (raw/ norm)
1	GLI (0.00400/ 1.000)	cos_doy (1.344/ 1.000)	GLI (0.0882/ 1.000)
2	wind_speed (0.00353/ 0.724)	GLI (0.680/ 0.488)	wind_speed (0.0688/ 0.755)
3	GVMI (0.00352/ 0.718)	wind_speed (0.470/ 0.327)	pressure (0.0580/ 0.619)
4	cos_doy (0.00352/ 0.718)	carbon_monoxide (0.281/ 0.181)	cos_doy (0.0493/ 0.509)
5	pressure (0.00310/ 0.471)	pressure (0.205/ 0.122)	NDVI (0.0362/ 0.343)
6	GARI (0.00304/ 0.435)	GVMI (0.194/ 0.114)	carbon_monoxide (0.0273/ 0.231)
7	nightlight_avg_rad (0.00254/ 0.141)	humidity (0.083/ 0.029)	GARI (0.0172/ 0.104)
8	sulphur_dioxide (0.00247/ 0.100)	temperature (0.062/ 0.012)	GVMI (0.0120/ 0.038)
9	NDVI (0.00245/ 0.088)	GARI (0.046/ 0.000)	wind_direction (0.0116/ 0.033)
10	carbon_monoxide (0.00230/ 0.000)	NDVI (0.046/ 0.000)	ozone (0.0090/ 0.000)

a.Gradient×Input and SHAP values are unitless importance scores; PFI is reported as MAE increase in µg/m^3^.

**Fig 8:**
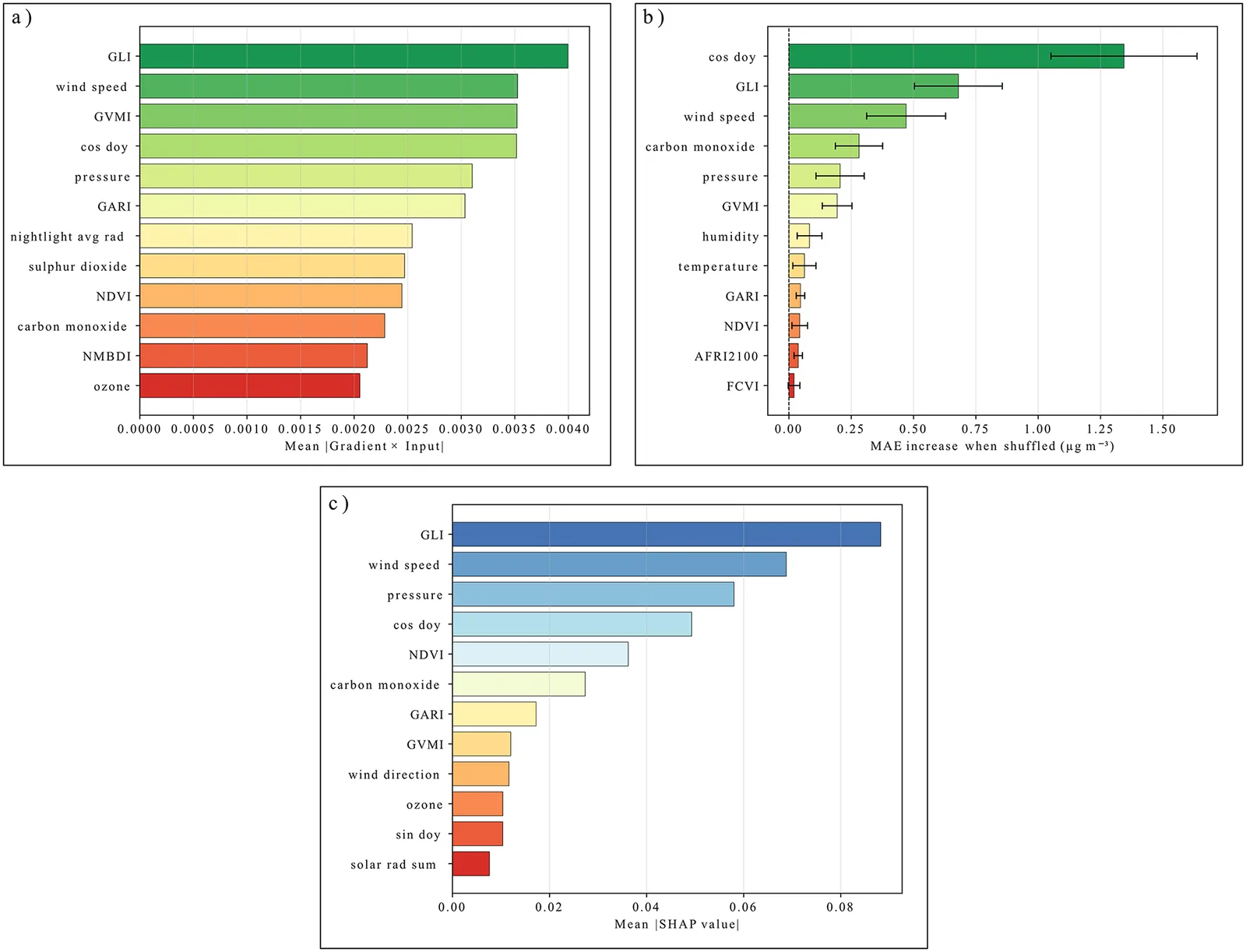
Feature importance rankings from three independent attribution methods. (a) Gradient × Input, (b) Permutation Feature Importance, and (c) KernelSHAP, demonstrating convergence on a consistent core of dominant predictors.

The very strong importance of cos_doy in PFI (Fig 8b) (MAE increase = 1.344, nearly double the second-ranked GLI at 0.680) compared with its lower ranking in Gradient × Input (4th) and SHAP (4th) shows an important difference between methods. When the seasonal encoding is permuted, the model’s seasonal pattern is strongly disrupted. In contrast, gradient and SHAP methods evaluate importance around individual samples, so they rank immediate meteorological variables higher for single predictions. This suggests that cos_doy mainly acts as a global seasonal structure in the model rather than a direct short-term predictor. The SHAP beeswarm plot (Fig 9) shows the direction and consistency of each feature’s effect on the + 1-day forecast. High GLI values (red points) appear mainly on the positive side of the axis, indicating that stronger vegetation greenness increases predicted PM_2.5_. This pattern follows Bangladesh’s agricultural calendar, where high vegetation greenness and residue burning during the post-harvest and pre-monsoon period contribute to increased pollution [8]. High wind_speed values produce mostly negative SHAP contributions, confirming their role in pollutant dispersion. At the daily scale, stronger winds generally reduce PM_2.5_ concentrations through enhanced mixing and dilution, although long-range transport may occasionally influence this relationship [48]. High atmospheric pressure also shifts predictions upward, consistent with stable anticyclonic conditions that suppress vertical mixing and trap pollutants near the surface [48].

**Fig 9:**
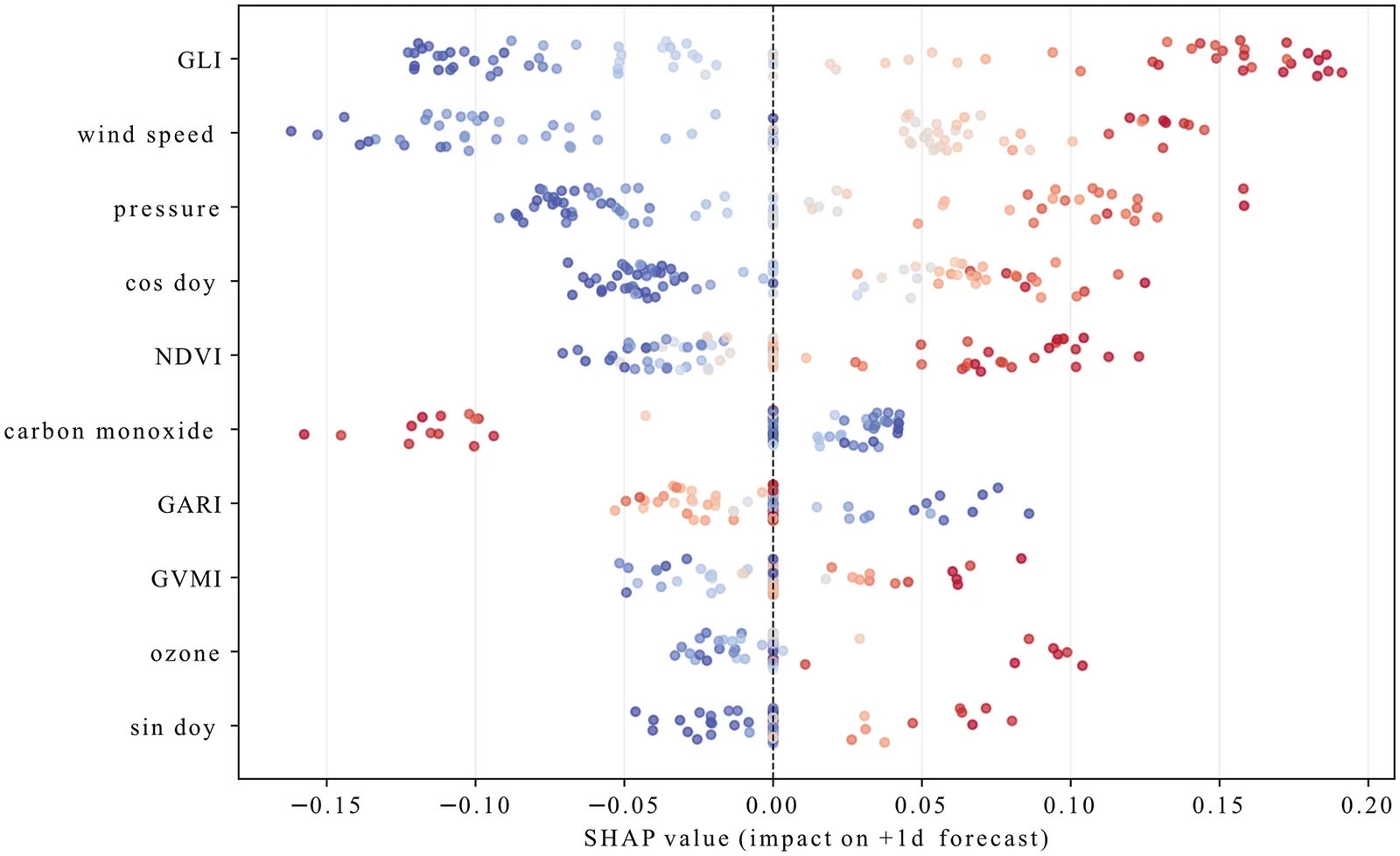
KernelSHAP beeswarm plot for the + 1-day forecast. It shows the direction and magnitude of each feature’s contribution; dot color indicates feature value (red = high, blue = low).

High CO values tend to produce negative SHAP contributions, likely reflecting complex feature interactions or proxy effects, rather than a direct causal relationship in which CO reduces PM_2.5_ [78]. The CO–PM_2.5._ relationship also varies seasonally, becoming weaker in warmer months when photochemical processes drive particle formation [79]. The cos_doy feature shows points on both sides of the axis without a clear direction, indicating that seasonal encoding mainly provides background temporal context rather than directly increasing or decreasing predictions for individual observations.

### Per-city feature heterogeneity

The city-level Gradient × Input heatmap at the + 1-day horizon (Fig 10) indicates substantial spatial variation in feature importance across the 12 cities. Dhaka’s second most important feature is nightlight_avg_rad (row-normalized value = 0.99, behind only GVMI at 1.00), reflecting its role as the country’s primary urban and industrial center where anthropogenic emissions strongly influence PM_2.5_ variability. In Sylhet, the feature profile is dominated by GLI (1.00) and GARI (0.92), consistent with its location in the northeastern tea-growing highland region where vegetation cycles and agricultural land use strongly affect particulate concentrations. The southwestern coastal cities, including Barisal, Khulna, and Jashore, show higher importance for GVMI (1.00). This index captures moisture conditions in vegetation and the boundary layer, which are influenced by maritime airflow from the Bay of Bengal in this region [31]. In the northwest, Bogra and Rajshahi are most sensitive to GLI and wind_speed. This pattern is consistent with their exposure to dry-season pollution transport from the Indo-Gangetic Plain, where northwesterly winds carry PM_2.5_ from major emission areas in northern India [4].

**Fig 10:**
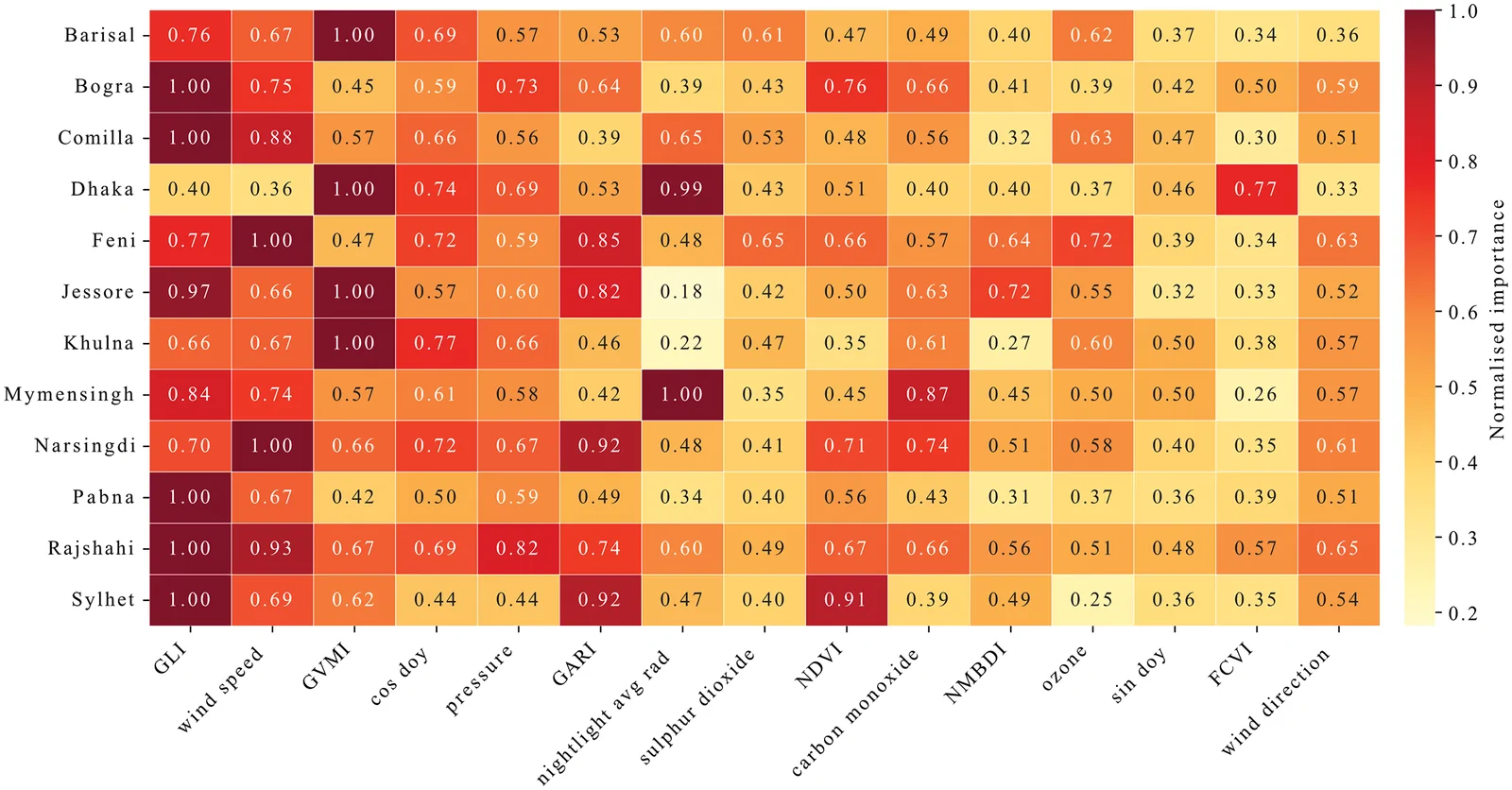
Row-normalized Gradient × Input feature importance heatmap at the + 1-day horizon.

### Temporal memory structure

The temporal sensitivity analysis (Fig 11a) estimates how far back in time the encoder effectively uses past information for prediction. The normalized gradient norm remains close to zero from t − 21 to t − 9 for all forecast horizons. This indicates that the model gains very little information from observations older than about 9–10 days. Sensitivity increases quickly from t − 8 and rises sharply toward t − 1, where the most recent observation provides the strongest signal for prediction. Fig 11b also shows a horizon-dependent crossover pattern. In the period between t − 6 and t − 3, the sensitivity for the + 3-day and +2-day forecasts becomes higher than that for the + 1-day forecast. This means that longer-range forecasts rely slightly more on observations from several days earlier. In contrast, the + 3-day forecast draws more information from earlier days to capture slower meteorological processes such as pressure changes and wind shifts that develop over several days and influence pollution conditions later. Overall, the sensitivity is concentrated within the most recent 5–6 days for all forecast horizons. This suggests that the 21-day lookback window is not fully used by the model.

**Fig 11:**
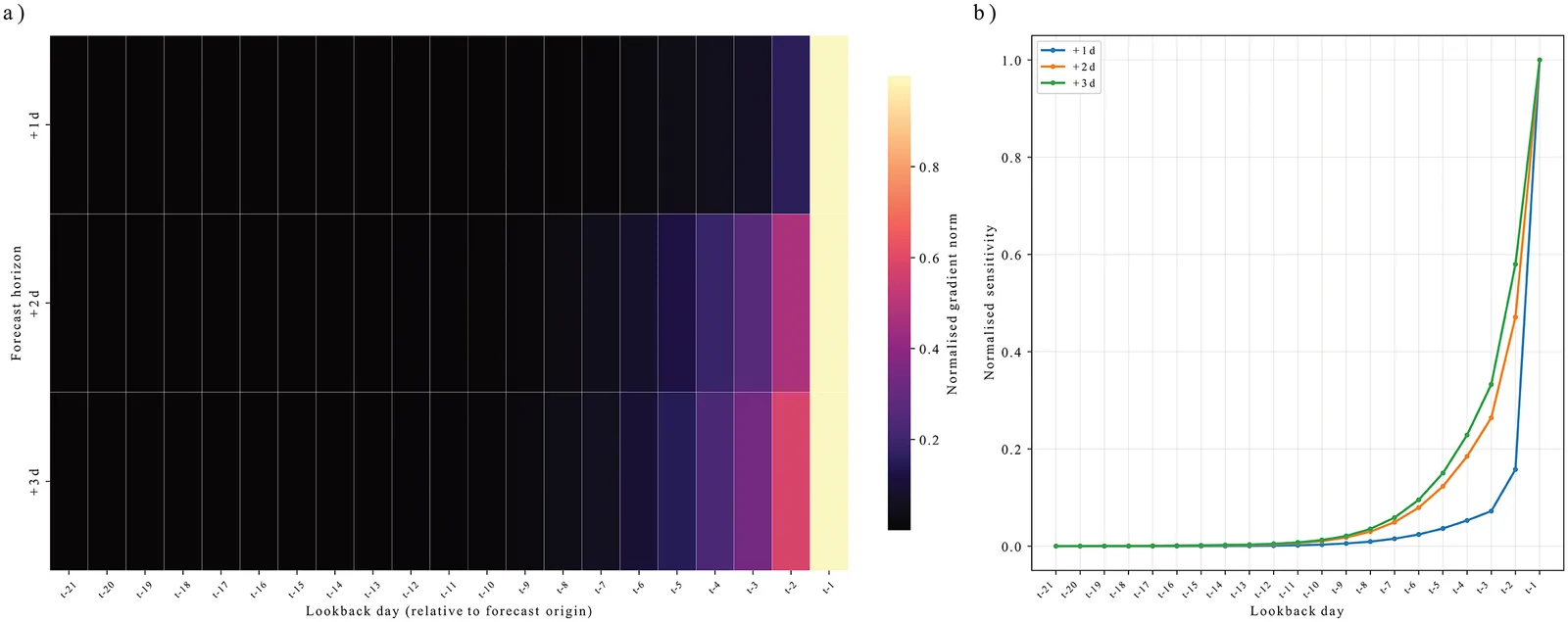
Temporal sensitivity of the GD-GRU encoder to lookback day. (a) Horizon × time heatmap of normalized gradient norm. (b) Horizon-separated line plot of the same normalized gradient norm.

### Spatial influence and graph topology validation

The occlusion-based spatial influence matrix (Fig 12a) measures how much the + 1-day forecast for each city changes when the full feature sequence of another city is masked. The diagonal values are much larger than the off-diagonal values, ranging from 0.26 to 0.44. This shows that each city’s forecast depends mainly on its own historical data, which is expected because the model produces separate outputs for each city. The off-diagonal values represent real cross-city influence learned through the graph diffusion mechanism. The strongest cross-city influences are Khulna → Jashore (0.054), Jashore → Khulna (0.053), Feni → Comilla (0.052), and Bogra → Rajshahi (0.052). These city pairs are geographically close and lie along known air transport pathways. In contrast, Mymensingh has a relatively weak diagonal value (0.26 compared with a network mean of about 0.38). This is consistent with its lower prediction performance (R^2^ = 0.529) and suggests that some pollution sources affecting this city are not represented in the current 12-city network.

**Fig 12:**
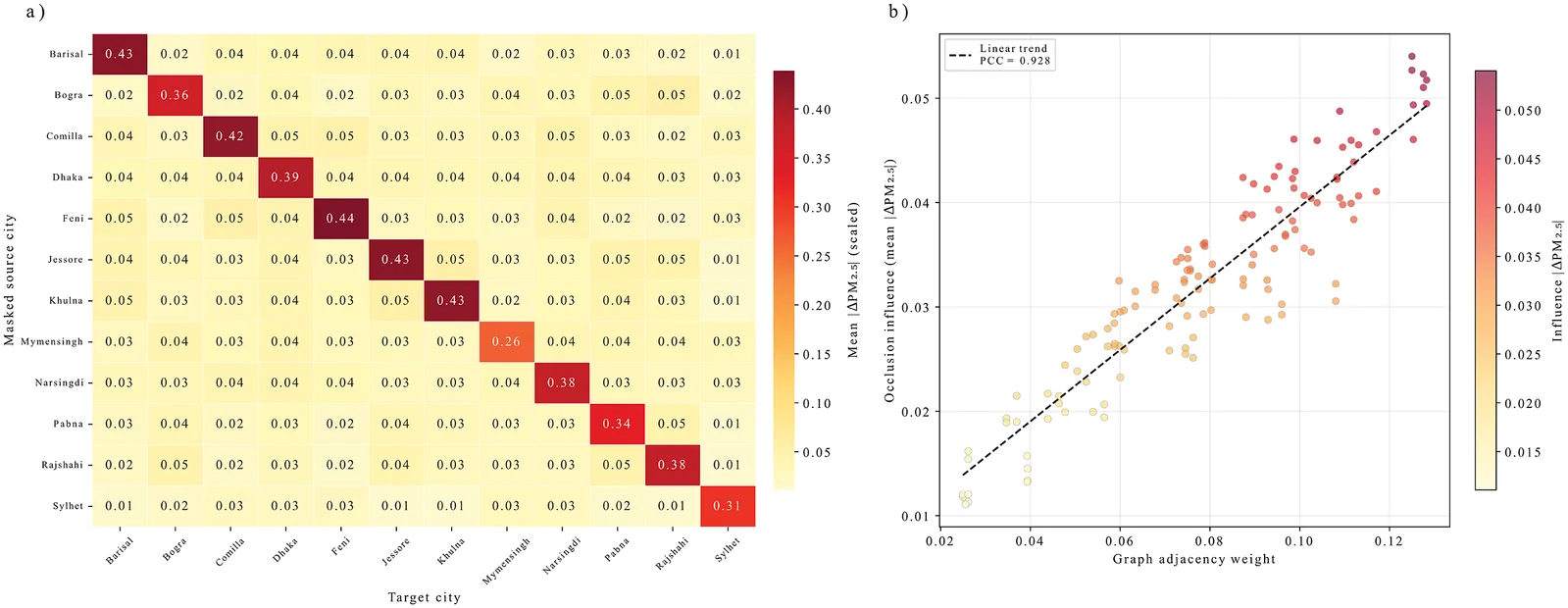
Occlusion-based spatial influence matrix and its agreement with geographic adjacency. (a) Spatial influence matrix Ω at the + 1-day horizon. (b) Scatter plot of off-diagonal Ω entries against geographic adjacency weights from Ã; Pearson r = 0.928 (p < 0.001).

Sylhet has the lowest average outgoing influence among all cities (0.0206), which is about 38% below the network mean (0.033). This indicates that Sylhet is relatively isolated in terms of pollution influence on other cities. This pattern is geographically reasonable because Sylhet is located in the far northeast and is separated from major population centers by hilly terrain and river floodplains that limit pollution transport. Sylhet also has one of the lowest self-diagonal values (0.31), suggesting that its own past conditions provide less predictive information than in most other cities. This matches the relatively low R^2^ value (0.534) observed for this city, even though its absolute prediction error remains small.

The key graph validation result is shown in Fig 12b. The Pearson correlation between the off-diagonal values of the learned occlusion influence matrix Ω and those of the fixed geographic adjacency matrix Ã is very high, with r = 0.928 (p < 0.001). This strong correlation provides clear empirical support for the geographic adjacency design. It shows that the city pairs where the model learns strong information flow are almost the same pairs that receive high weights in the distance-based Gaussian adjacency matrix. Importantly, the model was not trained to match this adjacency structure, so the agreement emerges independently from the learning process. The findings here suggest that a carefully designed static geographic graph, built from distance relationships and improved with domain knowledge, can serve as a reliable and interpretable approximation of the real atmospheric influence network.

### Sensitivity analysis of graph hyperparameters

To evaluate the robustness of heuristic choices in the graph construction pipeline, we conducted a sensitivity analysis on the Gaussian kernel bandwidth (σ) and stochastic edge-dropout rate (p). Bandwidth was varied as a multiplier on the default value (the median pairwise distance), from 0.5× to 2.0 × . Overall test MAE varies by only 0.07 µg/m^3^ and R^2^ by 0.006 across this four-fold range (S2 Table), indicating the model is not sensitive to precise tuning of σ. Edge-dropout rate was varied from 0.0 to 0.30; overall MAE varies by only 0.022 µg/m^3^ and R^2^ by 0.001 across this range (S3 Table), with a rate of 0.15 achieving the lowest MAE and the adopted rate of 0.10 performing within 0.003 µg/m^3^ of it. Both parameters can therefore be treated as low-risk defaults rather than hyperparameters requiring extensive tuning.

To evaluate the fixed geographic graph against adaptive and meteorologically informed alternatives, the five-seed GD-GRU ensemble was retrained under four alternative graph constructions: a k-nearest-neighbor graph, a wind-informed directional graph (where edge weight is scaled by the alignment between prevailing wind direction and inter-city bearing), a hybrid geographic-wind graph (convex combination with β = 0.5), and a fully learnable adaptive adjacency regularized with an L1 sparsity penalty to discourage overfitting in this 12-node network. All variants shared the same data split, training protocol, and ensemble size as the main model. As shown in S4 Table, no alternative construction improved the overall R^2^ or RMSE relative to the adopted geographic graph. The fully learnable adaptive adjacency achieved the lowest MAPE but the worst R^2^ and RMSE among all tested variants, which is consistent with an unconstrained learnable graph being prone to overfitting spatial correlations at this limited network size. Furthermore, the wind-informed and hybrid geographic-wind graphs performed within the margin of measurement noise of the adopted graph, indicating that incorporating directional meteorological information did not yield a measurable predictive improvement in this setting.

To validate the teacher-forcing schedule, we evaluated the decay length and initial ratio ($\rho_{\text{0}}$) by retraining the five-seed ensemble across multiple configurations. Decaying over the full 300-epoch budget outperformed shorter, fixed decay windows by 0.015–0.073 µg/m^3^ in MAE (S5 Table), demonstrating the benefit of a prolonged transition into fully autoregressive training prior to checkpoint selection. When evaluating initial ratios, disabling teacher forcing entirely ($\rho_{\text{0}}=\text{0}.\text{0}$) yielded the poorest performance (MAE 8.970 µg/m^3^). Ratios of 0.3, 0.6, and 0.9 performed within a narrow, statistically indistinguishable band of 0.006 µg/m^3^ (S6 Table), confirming the robustness of the adopted $\rho_{\text{0}}=\text{0}.\text{6}$. While higher initial ratios naturally required more epochs to converge (up to 136 epochs for $\rho_{\text{0}}=\text{0}.\text{9}$ versus 119 for $\rho_{\text{0}}=\text{0}.\text{3}$), the training dynamics remained stable.

Furthermore, the temporal sensitivity analysis indicated that effective gradient sensitivity is concentrated in the most recent 5–10 days of the 21-day lookback window. To validate this observation, we conducted a direct ablation by retraining the five-seed ensemble with lookback windows of 7, 10, 14, and 21 days, holding all other settings constant (S7 Table). A 10-day lookback achieved an R^2^ of 0.667, marginally outperforming the 21-day baseline (R^2^ = 0.666). Even a 7-day lookback successfully recovered an R^2^ of 0.663, performing within 0.5% of the 21-day result. These findings confirm that a substantially shorter lookback window (e.g., 7–10 days) captures the necessary temporal dependencies and achieves comparable predictive performance at a reduced computational cost.

## Discussion

### The geographic adjacency

The most important methodological finding of this study is the very high Pearson correlation (r = 0.928, p < 0.001) between the off-diagonal elements of the learned occlusion influence matrix Ω and the fixed geographic adjacency matrix A.

The GD-GRU model was trained only to minimize forecast error. It was not given any supervision to reproduce the geographic adjacency structure. Therefore, the spatial influence pattern that appears in the learned representation is an independent outcome of the training process. The very strong agreement between this learned structure and the Gaussian distance-based adjacency is consistent with geographic proximity being a major factor shaping inter-city PM_2.5_ influence in Bangladesh. Because this analysis is correlational rather than interventional, it cannot establish geographic proximity as a causal mechanism; it demonstrates only that a distance-based representation of space aligns closely with the spatial dependencies the model has learned from data. The strongest cross-city influences, Khulna→Jashore (0.054), Jashore→Khulna (0.053), Feni→Comilla (0.052), and Bogra→Rajshahi (0.052), occur between geographically closest city pairs and are consistent with known regional airflow pathways. The Khulna–Jashore corridor lies across the southwestern plain where westerly airflow is common. The Feni–Comilla pair is located along a southeastern valley pathway. The Bogra–Rajshahi pair lies within the northwestern agricultural belt that often receives pollution transported from the Indo-Gangetic Plain during the dry season [4,31].

This result also has practical importance for environmental forecasting in data-limited regions. When training data are scarce, fully learnable graph structures, such as adaptive graph convolution networks or attention-based spatial models, can easily overfit spatial relationships in small datasets. In such cases, the learned adjacency may reflect noise in the training data rather than real atmospheric processes [22]. The findings here suggest that a carefully constructed static geographic graph, supported by knowledge of terrain and regional airflow, can provide a reliable and interpretable representation of spatial influence in a small network. This offers a practical alternative to dynamic graph learning for developing countries with sparse monitoring networks.

### Overall predictive performance in data-scarce cities

The GD-GRU ensemble achieves an overall R^2^ of 0.666 and MAE of 8.853 μg/m^3^ across 12 cities and three forecast horizons, outperforming all baseline models, including the GRU without graph diffusion. These results should be considered in the context of Bangladesh’s monitoring conditions. In 2023, Bangladesh recorded an annual average PM_2.5_ concentration of 79.9 μg/m^3^, the highest among all countries and territories in the IQAir World Air Quality Report. Despite this severe pollution burden, the country operates one of the sparsest regulatory monitoring networks in South Asia. Only 12 stations were available for this study, producing about 822 training windows. This is far smaller than datasets used in similar studies from China, the United States, or Europe, where monitoring networks include hundreds or even thousands of stations [13]. The fact that useful multi-step forecasts can still be produced under these conditions is therefore an important finding for low-income countries where dense monitoring networks are unlikely in the near future.

The + 1-day R^2^ of 0.768 is broadly comparable to short-range GNN forecasting results reported in other South Asian studies. For example [80], reported R^2^ = 0.75 for a Spatially Attentive Cluster-based GNN applied to Delhi. That study used a much denser monitoring network than the one available in Bangladesh. This comparison suggests that the GD-GRU framework provides competitive predictive skill even under stronger data limitations. The improvement of the graph-based model over the GRU baseline without a graph component is modest but consistent. The smaller gain observed here is mainly due to the limited number of nodes, only 12 cities, and the weaker spatial signal available between them. Even so, the graph component improves all evaluation metrics across every ensemble seed. In addition, the occlusion-based spatial influence analysis shows that cross-city information flow exists and follows physically meaningful patterns, even though its overall contribution is constrained by the small 12-node network.

GD-GRU’s statistically significant advantage over DCRNN when pooled across forecast horizons (FDR-corrected DM test p < 0.001), combined with closely matched performance at each individual horizon, is an interpretable and favorable result rather than a limitation. Both architectures use the same training pipeline, regularization, and graph structure. Their similar performance suggests that in this 12-node, data-limited setting, the choice between Chebyshev polynomial approximation and bidirectional random-walk diffusion is less influential than shared design choices such as fixed geographic adjacency, ensemble averaging, and the regularization setup [14,51]. This supports the paper’s central argument that graph construction may matter more than the diffusion operator in sparse monitoring networks [22]. GD-GRU remains preferable in practice because it operates directly on the symmetric normalized adjacency without requiring separate forward and backward transition matrices, preserves the spectral properties of the graph Laplacian [51], and trains about 40% faster than DCRNN. The temporal sensitivity analysis reveals a physically interpretable pattern. Between t − 6 and t − 3, gradient sensitivity for the + 3-day and +2-day forecasts is higher than for the + 1-day forecast, indicating that longer-range predictions rely more on observations from several days earlier to capture slower atmospheric processes, such as pressure system changes and shifts in wind patterns [49,81]. In Bangladesh, this includes dry-season pollution build-up under stable northerly winds and pressure-driven transitions during the pre-monsoon period [4,31]. This multi-scale temporal behavior shows that the GD-GRU encoder captures atmospheric memory at multiple time scales, even though most of its sensitivity is concentrated within the most recent five to six days.

### Per-city heterogeneity and environmental mechanisms

The large variation in per-city R^2^, ranging from 0.529 in Mymensingh to 0.709 in Khulna, reflects real differences in pollution environments across Bangladesh’s cities. The southern coastal cities, including Barisal, Khulna, and Jashore, show the strongest predictive performance. These cities are influenced by maritime airflow from the Bay of Bengal, which creates relatively stable and seasonally consistent boundary-layer conditions [31,48].

The strong importance of GVMI in their feature profiles supports this explanation. Moisture in vegetation and the boundary layer, which is strongly affected by sea conditions and onshore moisture transport, becomes a key factor controlling PM_2.5_ levels in this region. The north-central cities, including Bogra, Mymensingh, and Rajshahi, show weaker performance. The weaker performance in north-central cities is due to three main factors. First, pollution transported from the Indo-Gangetic Plain causes sudden PM_2.5_ spikes during the dry season that the model cannot capture [4,8]. Second, emissions from brick kilns create sharp peaks that are not well represented by daily meteorological data [5,82]. Third, mixed land use, including brick kilns, crop burning, and peri-urban growth, introduces local variability beyond what the 12-city network can represent. Including wind data from the Indo-Gangetic Plain and more detailed emission information could improve predictions. Sylhet’s performance can be explained by the structure of pollution variability in the city. Sylhet lies in a northeastern highland area, bordered by the Khasi Hills and Jaintia Hills. These hills partly block pollution transport from the Indo-Gangetic Plain and reduce overall PM_2.5_ variability compared with other cities in the network [31]. When pollution levels vary less, a model that predicts values close to the average can achieve low absolute error but still explain little variance. This results in low R^2^ but low MAE at the same time. Mymensingh is a clear exception to the overall spatial pattern. Its self-diagonal value in Ω (0.26) is much lower than the network average (0.38), which means the model relies less on its own historical data than for other cities. This pattern suggests that important drivers of pollution in Mymensingh come from outside the 12-city network. The combination of low self-influence and weaker predictive performance for Mymensingh therefore indicates that part of its PM_2.5_ variability is driven by external factors that are not represented in the current network.

### Feature importance and atmospheric seasonality

Gradient × Input, Permutation Feature Importance (PFI), and KernelSHAP consistently highlight GLI, wind_speed, pressure, and cos_doy as the dominant features. This convergence across methods with different underlying principles supports the robustness of these features as key drivers of the model’s behavior [26,28]. GLI ranks above NDVI consistently across all three attribution methods, and the reason lies in how the two indices behave under dense vegetation conditions. Both indices measure vegetation greenness, but NDVI saturates under dense canopy conditions, particularly in Bangladesh’s floodplain and delta regions where monsoon vegetation keeps NDVI near its ceiling for several months [83]. GLI incorporates green, red, and blue reflectance bands and avoids this saturation, remaining sensitive across the full agricultural cycle including planting, harvest, and fallow periods. These cycles directly drive crop residue burning, land preparation, and surface roughness changes that influence PM_2.5_ formation and dispersion [8]. GLI therefore captures seasonally relevant land surface signals that NDVI cannot, which explains its consistently higher importance across all three attribution methods. The difference between the ranking of cos_doy in PFI (first, with an MAE increase of 1.344 μg/m^3^) and its ranking in Gradient × Input and SHAP (fourth in both) shows that seasonal encoding plays a different role in the model. The much larger impact seen in PFI suggests that cos_doy mainly provides the overall seasonal structure for the model. It helps the model understand where each observation falls within the yearly cycle rather than strongly affecting short-term predictions [28].

The directional SHAP analysis further shows that the four main features influence predictions through different physical processes. GLI has a clear positive relationship with predicted PM_2.5_, while wind_speed shows a consistent negative relationship. Both effects appear stable across most samples, indicating relatively steady and near-linear behavior at the daily scale. Atmospheric pressure shows a wide range of SHAP values, indicating that its effect on PM_2.5_ depends on other factors such as temperature stratification and wind patterns. In contrast, cos_doy has a balanced spread on both sides of the axis, suggesting it mainly provides seasonal context without a consistent effect on individual predictions.

### Limitations

Several limitations of this study should be noted. First, the 12-city network represents the key urban centers of Bangladesh, but large parts of the country remain unmonitored. The anomaly observed in Mymensingh suggests that external pollution sources affect the network. In particular, emissions from the Indo-Gangetic Plain and the Meghalaya highlands can influence northern Bangladesh, but these sources are outside the current network and cannot be represented directly by the model [4,8]. One possible solution is to include additional inputs from global atmospheric datasets. Second, the model uses daily averaged PM_2.5_ concentrations. This means it cannot capture diurnal variation, such as morning and evening peaks caused by boundary-layer changes and traffic emissions. Predicting these short-term variations would require higher-frequency monitoring data, which are currently unavailable at most stations in Bangladesh. However, the GD-GRU architecture can easily work with higher-resolution inputs, so this limitation mainly reflects the current data availability. As monitoring improves through programs such as the Bangladesh Department of Environment Continuous Air Monitoring Station initiative, the model could use sub-daily data without major architectural changes. Third, the static geographic adjacency used in the graph structure does not account for seasonal changes in wind direction or monsoon-driven shifts in atmospheric circulation. Although our testing demonstrated that simple wind-informed hybrid weighting did not improve predictive performance, developing dynamic spatiotemporal graphs that capture complex seasonal circulation shifts without overfitting remains an important area for future research. Fourth, in addition to missing external sources, the small size of the 12-node network inherently constrains the available spatial signal and increases the risk of overfitting flexible spatial relationships. Our results show that a fully learnable adaptive adjacency performed worse than the fixed geographic graph, reinforcing the value of domain-informed structure in sparse networks. In addition, the gap-filling methods, including linear interpolation and forward-filling satellite composites, may introduce temporal bias by smoothing brief pollution spikes or masking rapid agricultural changes, while the temporal collapse required by KernelSHAP and permutation methods limits their ability to resolve time-lag effects. Although agreement with gradient-based scores suggests that overall feature rankings remain robust, graph- and time-aware methods such as GraphSHAP [84] and GraphSVX [70] provide a more principled direction for future attribution analysis. Finally, this study has not been externally validated against monitoring networks outside Bangladesh. Cross-region testing, for example against other data-scarce South Asian networks, would help establish whether the reported forecasting performance and the geographic-graph validation findings generalize beyond this specific 12-city network.

## Conclusion

This study demonstrates that reliable multi-city, multi-step PM_2.5_ forecasting is possible under monitoring conditions that are far more data-scarce than those used in comparable spatiotemporal GNN studies. A Chebyshev graph diffusion GRU ensemble is applied to Bangladesh’s 12-city network, which is among the sparsest regulatory air quality networks in South Asia. The study addresses four key questions: whether a static geographic graph can approximate learned spatial influence patterns; whether useful multi-step forecasts can be achieved in such a sparse setting; which features consistently drive predictions across different attribution methods; and whether the choice of diffusion operator is important under limited data conditions. The GD-GRU ensemble achieves an overall R^2^ of 0.666 and an MAE of 8.853 μg/m^3^. The + 1-day forecast reaches an R^2^ of 0.768, which is broadly comparable to results reported for denser monitoring networks. The model significantly outperforms all non-graph baselines (p < 0.001), confirming the value of graph diffusion even in a 12-node network. Comparison with DCRNN shows closely matched performance at each individual forecast horizon, with only a negligible-effect-size difference detected when pooling across horizons (Cohen’s d = −0.041), favoring GD-GRU. This suggests that in sparse networks, graph construction and regularization design contribute more to forecasting performance than the specific choice of diffusion operator. GD-GRU remains preferable in practice due to its symmetric adjacency formulation, spectral consistency, and approximately 40% faster training.

A key methodological finding is the strong Pearson correlation (r = 0.928, p < 0.001) between the learned occlusion influence matrix and the fixed geographic adjacency. This agreement emerges solely from minimizing forecast error, without any supervision toward the adjacency structure. This correlational agreement supports the use of a static distance-based graph as a practical approximation of atmospheric influence patterns in data-limited settings. It offers a practical alternative to adaptive graph learning, which may overfit when training data are limited. The explainability analysis further identifies GLI, wind speed, atmospheric pressure, and seasonal encoding as consistently important predictors. Regional feature patterns are physically interpretable: moisture-related variables dominate in southern coastal cities, while vegetation and wind features are more influential in northwestern cities affected by Indo-Gangetic Plain transport.

Several limitations should be noted. The model relies on daily averages, which may miss sub-daily variability. The static graph cannot capture seasonal changes in atmospheric circulation. The 21-day lookback window is not fully utilized. External pollution sources are not fully represented, particularly for Mymensingh, where the low self-influence value suggests that important drivers lie outside the current network. As monitoring infrastructure in Bangladesh expands, the GD-GRU framework can incorporate higher temporal resolution data and additional nodes without requiring structural changes. Overall, this study shows that interpretable and geographically grounded spatiotemporal GNN forecasting is feasible in highly data-scarce environments, and that static geographic graphs can serve as reliable spatial representations in similar developing-country contexts.

### Declaration of generative AI and AI-assisted technologies in the writing process

During the preparation of this manuscript, the author used Claude (Anthropic) to improve the language, clarity, readability, and formatting of the text. Following the use of this tool, the author carefully reviewed and edited all outputs to ensure accuracy, scientific integrity, and alignment with the study’s original findings. The author takes full responsibility for the content of this publication. Generative AI was not used for any aspect of the research design, data collection, data analysis, results interpretation, or the formulation of conclusions. No AI-generated images, figures, or tables are included in this manuscript.

## Supporting information

S1 Table Cross-method agreement in feature attribution (12-city test set, + 1-day horizon). (PDF)

S2 Table Sensitivity to Gaussian kernel bandwidth (σ = multiplier × median pairwise distance). (PDF)

S3 Table Sensitivity to stochastic edge-dropout rate (5-seed ensemble). (PDF)

S4 Table Overall test performance under alternative graph constructions (5-seed ensembles). (PDF)

S5 Table Sensitivity to teacher-forcing decay length (5-seed ensemble; ρ₀ = 0.6). (PDF)

S6 Table Sensitivity to initial teacher-forcing ratio ρ₀ (5-seed ensemble; decay over full budget). (PDF)

S7 Table Overall test set performance by lookback window length (5-seed ensemble). (PDF)
