# Supplementary material for: Explainable spatiotemporal Graph Neural Networks: Multi-City PM2.5 forecasting in Bangladesh with geographic graph topology validation
Source: PLoS One. 2026 Oct 12;21(10):e0351928. doi: 10.1371/journal.pone.0351928 (PMC13657321; doi:10.1371/journal.pone.0351928)
Supplement: Supplementary file 1 [file pone.0351928.s001.pdf]

S1 Table. Cross-method agreement in feature attribution (12-city test set, +1-day horizon).

Spearman rank correlation (ρ), with p-values in parentheses

	**Gradient × Input**	**PFI**	**SHAP**
**Gradient × Input**	—	0.590 (0.0019)	0.783 (3.7×10^{-6})
**PFI**	0.590 (0.0019)	—	0.678 (1.98×10^{-4})
**SHAP**	0.783 (3.7×10^{-6})	0.678 (1.98×10^{-4})	—

Top-10 feature-set Jaccard overlap

	**Gradient × Input**	**PFI**	**SHAP**
**Gradient × Input**	1.000	0.667	0.667
**PFI**	0.667	1.000	0.538
**SHAP**	0.667	0.538	1.000
