# Supplementary material for: Explainable spatiotemporal Graph Neural Networks: Multi-City PM2.5 forecasting in Bangladesh with geographic graph topology validation
Source: PLoS One. 2026 Oct 12;21(10):e0351928. doi: 10.1371/journal.pone.0351928 (PMC13657321; doi:10.1371/journal.pone.0351928)
Supplement: Supplementary file 2 [file pone.0351928.s002.pdf]

S2a Table. Sensitivity to Gaussian kernel bandwidth (σ = multiplier × median pairwise distance).

σ multiplier	MAE	RMSE	MAPE (%)	R^2	PCC
0.50	8.861	12.195	33.75	0.6633	0.8184
0.75	8.901	12.186	34.56	0.6638	0.8169
1.00 (adopted)	8.865	12.095	34.53	0.6689	0.8195
1.25	8.850	12.082	34.49	0.6696	0.8199
1.50	8.838	12.116	34.06	0.6677	0.8194
2.00	8.828	12.105	34.03	0.6683	0.8197
