# Supplementary material for: Explainable spatiotemporal Graph Neural Networks: Multi-City PM2.5 forecasting in Bangladesh with geographic graph topology validation
Source: PLoS One. 2026 Oct 12;21(10):e0351928. doi: 10.1371/journal.pone.0351928 (PMC13657321; doi:10.1371/journal.pone.0351928)
Supplement: Supplementary file 3 [file pone.0351928.s003.pdf]

S2b Table. Sensitivity to stochastic edge-dropout rate (5-seed ensemble).

Edge-dropout rate	MAE	RMSE	MAPE (%)	R^2	PCC	Stopping epoch (mean [min–max])
0.00	8.872	12.122	34.96	0.6674	0.8183	119.4 [119–121]
0.05	8.855	12.138	34.17	0.6665	0.8187	120.2 [119–125]
0.10 (adopted)	8.853	12.139	34.13	0.6664	0.8187	120.2 [119–125]
0.15	8.850	12.133	34.11	0.6668	0.8189	120.2 [119–125]
0.20	8.861	12.143	34.28	0.6662	0.8184	120.2 [119–125]
0.30	8.860	12.150	34.09	0.6658	0.8186	121.2 [119–125]
