# Supplementary material for: Explainable spatiotemporal Graph Neural Networks: Multi-City PM2.5 forecasting in Bangladesh with geographic graph topology validation
Source: PLoS One. 2026 Oct 12;21(10):e0351928. doi: 10.1371/journal.pone.0351928 (PMC13657321; doi:10.1371/journal.pone.0351928)
Supplement: Supplementary file 4 [file pone.0351928.s004.pdf]

S3 Table. Overall test performance under alternative graph constructions (5-seed ensembles).

Graph construction	MAE	RMSE	MAPE (%)	R^2	PCC
Fixed geographic (adopted)	8.853	12.139	34.13	0.6664	0.8187
k-nearest-neighbor	8.896	12.174	35.02	0.6645	0.8166
Wind-informed (directional)	8.848	12.153	34.25	0.6657	0.8181
Hybrid geographic–wind ($\beta = 0.5$)	8.870	12.173	34.44	0.6645	0.8172
Fully learnable adaptive (L1-regularized)	8.881	12.302	33.00	0.6574	0.8176
