# Supplementary material for: Explainable spatiotemporal Graph Neural Networks: Multi-City PM2.5 forecasting in Bangladesh with geographic graph topology validation
Source: PLoS One. 2026 Oct 12;21(10):e0351928. doi: 10.1371/journal.pone.0351928 (PMC13657321; doi:10.1371/journal.pone.0351928)
Supplement: Supplementary file 5 [file pone.0351928.s005.pdf]

S4a Table. Sensitivity to teacher-forcing decay length (5-seed ensemble; $\rho_0 = 0.6$).

Decay length (epochs)	MAE	RMSE	MAPE (%)	R^2	PCC	Stopping epoch (mean [min–max])
50	8.926	12.217	34.62	0.6621	0.8162	119.0 [119–119]
100	8.889	12.213	34.15	0.6624	0.8170	120.4 [119–126]
150	8.888	12.192	34.30	0.6635	0.8172	120.8 [119–126]
200	8.867	12.164	34.22	0.6651	0.8179	120.2 [119–125]
300 (adopted; full budget)	8.853	12.139	34.13	0.6664	0.8187	120.2 [119–125]
