# Supplementary material for: Explainable spatiotemporal Graph Neural Networks: Multi-City PM2.5 forecasting in Bangladesh with geographic graph topology validation
Source: PLoS One. 2026 Oct 12;21(10):e0351928. doi: 10.1371/journal.pone.0351928 (PMC13657321; doi:10.1371/journal.pone.0351928)
Supplement: Supplementary file 6 [file pone.0351928.s006.pdf]

S4b Table. Sensitivity to initial teacher-forcing ratio ρ_0 (5-seed ensemble; decay over full budget).

Initial ratio ρ_0	MAE	RMSE	MAPE (%)	R^2	PCC	Stopping epoch (mean [min–max])
0.0	8.970	12.258	34.97	0.6599	0.8151	119.0 [119–119]
0.3	8.895	12.205	34.27	0.6628	0.8172	119.0 [119–119]
0.6 (adopted)	8.889	12.213	34.15	0.6624	0.8170	120.4 [119–126]
0.9	8.889	12.208	33.96	0.6626	0.8175	124.8 [119–136]
