# Supplementary material for: Explainable spatiotemporal Graph Neural Networks: Multi-City PM2.5 forecasting in Bangladesh with geographic graph topology validation
Source: PLoS One. 2026 Oct 12;21(10):e0351928. doi: 10.1371/journal.pone.0351928 (PMC13657321; doi:10.1371/journal.pone.0351928)
Supplement: Supplementary file 7 [file pone.0351928.s007.pdf]

S5 Table. Overall test set performance by lookback window length (5-seed ensemble).

Lookback (days)	Training windows	MAE	RMSE	MAPE (%)	R^2	PCC
7	836	8.992	12.362	34.11	0.6626	0.8161
10	833	8.926	12.289	34.18	0.6669	0.8185
14	829	8.834	12.012	35.22	0.6685	0.8180
21 (adopted)	822	8.853	12.139	34.13	0.6664	0.8187
